# Molecular docking, molecular dynamics simulations and binding free energy studies of interactions between *Mycobacterium tuberculosis* Pks13, PknG and bioactive constituents of extremophilic bacteria

**DOI:** 10.1038/s41598-024-57124-9

**Published:** 2024-03-21

**Authors:** Kudakwashe Nyambo, Kudzanai Ian Tapfuma, Francis Adu-Amankwaah, Lauren Julius, Lucinda Baatjies, Idah Sithole Niang, Liezel Smith, Krishna Kuben Govender, Mkhuseli Ngxande, Daniel J. Watson, Lubbe Wiesner, Vuyo Mavumengwana

**Affiliations:** 1 DST-NRF Centre of Excellence for Biomedical Tuberculosis Research; South African Medical Research Council Centre for Tuberculosis Research; Division of Molecular Biology and Human Genetics, Faculty of Medicine and Health Sciences, Stellenbosch University, Tygerberg, 7505 Cape Town, South Africa; 2 Department of Biotechnology and Biochemistry, University of Zimbabwe, B064, Mount Pleasant, Harare, Zimbabwe; 3 Computer Science Division, Department of Mathematical Sciences, Faculty of Science, University of Stellenbosch, Matieland, South Africa; 4 Department of Chemical Sciences, University of Johannesburg, Doornfontein Campus, P.O. Box 17011, Johannesburg, 2028 South Africa; 5 National Institute for Theoretical and Computational Sciences (NITheCS), Cape Town, South Africa; 6 Division of Clinical Pharmacology, Department of Medicine, Faculty of Health Sciences, University of Cape Town, Cape Town, South Africa

**Keywords:** Tuberculosis, Biotechnology, Molecular biology

## Abstract

Mycobacterial pathogens present a significant challenge to disease control efforts globally due to their inherent resistance to multiple antibiotics. The rise of drug-resistant strains of *Mycobacterium tuberculosis* has prompted an urgent need for innovative therapeutic solutions. One promising way to discover new tuberculosis drugs is by utilizing natural products from the vast biochemical space. Multidisciplinary methods can used to harness the bioactivity of these natural products. This study aimed to evaluate the antimycobacterial efficacy of functional crude extracts from bacteria isolated from gold mine tailings in South Africa. Bacterial strains were identified using 16S rRNA sequencing. The crude extracts obtained from the bacteria were tested against *Mycobacterium tuberculosis* H37Rv, *Mycobacterium smegmatis* mc^2^155, and *Mycobacterium aurum* A+. Untargeted HPLC-qTOF and molecular networking were used to identify the functional constituents present in extracts that exhibited inhibitory activity. A virtual screening workflow (VSW) was used to filter compounds that were strong binders to *Mycobacterium tuberculosis* Pks13 and PknG. The ligands returned from the VSW were subjected to optimization using density functional theory (DFT) at M06-2X/6-311++ (*d,p*) level of theory and basis set implemented in Gaussian16 Rev.C01. The optimized ligands were re-docked against *Mycobacterium tuberculosis* Pks13 and PknG. Molecular dynamics simulation and molecular mechanics generalized born surface area were used to evaluate the stability of the protein–ligand complexes formed by the identified hits. The hit that showed promising binding characteristics was virtually modified through multiple synthetic routes using reaction-driven enumeration. Three bacterial isolates showed significant activity against the two strains of *Mycobacterium*, while only two, *Bacillus subtilis* and *Bacillus licheniformis*, exhibited activity against both *Mycobacterium tuberculosis* H37Rv, *Mycobacterium smegmatis* mc^2^155, and *Mycobacterium aurum* A+. The tentatively identified compounds from the bacterial crude extracts belonged to various classes of natural compounds associated with antimicrobial activity. Two compounds, cyclo-(L-Pro-4-OH-L-Leu) and vazabitide A, showed strong binding against PknG and Pks13, with pre-MD MM-GBSA values of − 42.8 kcal/mol and − 47.6 kcal/mol, respectively. The DFT-optimized compounds exhibited the same docking scores as the ligands optimized using the OPSL-4 force field. After modifying vazabitide A, its affinity to the Pks13 binding site increased to − 85.8 kcal/mol, as revealed by the post-MD MM-GBSA analysis. This study highlights the potential of bacteria isolates from gold mine tailings as a source of new scaffolds for designing and optimizing anti-*Mycobacterium* agents. These agents synthesized in-silico can be further tested in-vitro to evaluate their efficacy.

## Introduction

Infectious diseases, such as tuberculosis (TB), have a significant negative impact globally and are a leading cause of death and disability. Despite the availability of treatment, TB still causes around 1.5 million deaths each year^1,2^. The current TB chemotherapies used in clinical practice are administered over a lengthy period, which can lead to poor patient compliance with treatment. *Mycobacterium tuberculosis* (*M.tb*) gene mutations, along with incomplete adherence to prolonged treatment regimens and co-infection with HIV, further exacerbate the emergence of drug-resistant strains. Therefore, it is essential to develop new and effective drugs to address the rapid increase in drug-resistant strains and reduce the duration of TB treatment^3^.

Some taxonomically different microorganisms within environmental niches do not amicably interact because of limited space and resources. Researchers have reported that microorganisms dominate in environmental niches through the strategic employment of diverse arsenal systems, for instance, strain-specific bacteriocins and broad-spectrum antimicrobials^4–6^. Understanding the mechanisms and driving forces that bacteria use in antagonistic interactions is crucial in the field of drug discovery. Studies have demonstrated that the *Bacillus* genus, which is found in soil, is a prolific source of a wide range of bioactive small molecules. These molecules include non-ribosomal cyclic lipopeptides, polyketides, and discoipyrole alkaloids, which possess antimicrobial properties. *Bacillus subtilis* and *B. licheniformis* have been reported to produce metabolites that can inhibit the growth of *Candida albicans*, *Heliobacter pylori*, and *M. tuberculosis*^7,8^. *Streptomycetes,* a type of filamentous actinobacteria frequently isolated from soil, have long been reported to be an immeasurable reservoir of novel metabolites, which account for two-thirds of agriculturally and medically essential secondary metabolites. Among these metabolites are some broad-spectrum antimicrobials such as streptomycin, ivermectin, nystatin, and tetracycline^9,10^. In a previous study, the efficacy of secondary metabolites from *Streptomyces* sp. against *M. tuberculosis* have been investigated^11^. The researchers revealed that the *Streptomyces* sp produces d-Cycloserine, which has been found to exhibit anti-*M. tuberculosis* activity by targeting the cell wall, d-alanine racemase (Alr), and d-alanine: d-alanine ligase (Ddl) at a minimum inhibitory concentration of (MIC = 14–900 μM). Another study revealed that pyridomycin, an antibacterial agent produced by *Streptomyces pyridomyceticus* demonstrated anti-*M. tuberculosis* activity at a MIC of 0.39 µg/ml^12^*.*

The extraction and processing of valuable minerals, such as gold, in the Gauteng province of South Africa have led to the creation of large mine tailings dumps. These dumps are recognized as a source of secondary environmental contamination. Gold mine tailings are typically characterized by scarce organic matter, low pH levels, and high concentrations of heavy metals^13,14^. Anthropogenic factors in gold mine tailings have exerted a detrimental impact on the ecosystem, especially the microbial communities, by acting as a selection pressure. However, it is interesting to note that the bacteria inhabiting the gold mine tailings have managed to adapt to these extreme conditions by modifying their metabolic and genomic processes to cope with the environmental stresses. Evidence from the whole genome analysis of *Serratia* sp. and *Stenotrophomonas* sp., which were isolated from waste-rock piles of abandoned gold mines, indicates genomic plasticity, driven by the acquisition of functional gene clusters via horizontal operon transfer^15^. Fundamentally, the transferred functional gene clusters enhance microbial survival fitness by expressing various unique metabolic pathways to extract energy from a wide array of inorganic electron donors and acceptors, thus compensating for the fluctuating, harsh gold mine tailings environments^16,17^. Another example is the spectacular phenotypic plasticity of some Actinobacteria, which elevates their potential to produce novel secondary metabolites in response to the fluctuating tailings environment. Therefore, gold mine tailings are a unique target for biomining a wide range of natural products that have a medical impact^18,19^.

A targeted and rational approach to TB drug discovery involves a comprehensive understanding of the pathogen at the molecular level, specifically the pathways involved in maintaining its fitness. This understanding help in identifying the druggable macromolecular targets or pathways that are critically involved in a disease state to solve the problem of XDR and MDR (Multi Drug Resistant) TB^20–23^. One of the essential components of *M. tuberculosis* survival and virulence is the serine-threonine protein kinase, PknG. This kinase regulates cell wall biosynthesis and cell division, making it an attractive macromolecular druggable target for drug development^24,25^. *Mycobacterium tuberculosis* Pks13 is responsible for producing mycolic acids that are critical building blocks of the bacterial cell wall. These mycolic acids are virulence factors that protect the bacterium from the host's immune defenses^26^. It is crucial to understand how inhibitors interact with Pks13 and PknG to develop new TB treatments.

Interestingly, the bioactive arsenal of extremophilic microorganisms has structurally evolved over time to optimize their endogenous and exogenous defence systems. As such, it is a prerequisite to identify and investigate the antimicrobial properties of the unique bioactive secondary metabolites that serve to govern bacterial communities through their extermination^27,28^. To date, there are currently no reports available regarding the mycobacterial activity of bioactive secondary metabolites originating from bacteria isolated from South African gold mine tailings. This suggests that such tailings represent an underexplored reservoir of bacterial genomes, which may hold considerable promise in terms of producing novel scaffolds with antimycobacterial activity. Harnessing the novel metabolic machinery of the extremophilic bacteria via metabolomics provides promising molecular starting points for the development of anti-TB drugs. Recent advancements in technology have accelerated the discovery of lead compounds and the design of potential drugs. During the hit-lead discovery phase of drug discovery, virtual screening, a computational method used to screen large libraries of compounds, is employed to identify molecular starting points or hits. Molecular dynamics (MD) simulations are one of the techniques used in virtual screening, allowing the comprehension of complex physical dynamics of a biological system at an atomic level, thus revealing the hidden states of a biological system that cannot be detected experimentally^29,30^. The aim of this study is to extract bacteria from tailings of a gold mine and to test their crude extracts for metabolites against *Mycobacterium smegmatis* mc^2^155, *Mycobacterium aurum* A+, and *M. tuberculosis* H37Rv. The chemical classes present in bacterial crude extract were tentatively identified using high-pressure liquid chromatography coupled to a quadrupole time-of-flight high-resolution mass spectrometer (HPLC-qTOF). Subsequently, molecular networking was conducted using the Global Natural Product Social platform. The compounds tentatively identified were then screened virtually against the essential proteins of *M. tuberculosis* H37Rv, i.e., Pks13 and PknG.

## Materials and methods

Gold mine tailings were acquired from five different sites in the Germiston in Johannesburg, South Africa (26° 13′ 7.08″ S, 28° 29′ 8.64″ E). The samples were collected from a depth of 12 cm, with each site providing 0.5 kg of sample material. The samples were then stored in polyethylene bags at 4 ℃ until analyzed.

### Isolation of bacteria

The culturable bacterial community available in the mine tailings samples was isolated by adding one gram of each soil sample to nine mL of sterile saline water (0.85% NaCl *w/v*) and thoroughly mixing it by vortexing. The upper suspension of each mixture was collected and serially diluted at a tenfold gradient. The bacteria were then cultivated by inoculating 50 µL of each diluted sample onto three different types of growth media: nutrient agar (N.A.), Luria–Bertani agar (L.B.), and tryptic soy agar (TSA)^31^. These three types of growth media were used to increase the likelihood of isolating a broad spectrum of culturable bacteria. The plates were incubated overnight at room temperature under aerobic conditions. Colonies were randomly picked and streaked onto fresh media plates to obtain pure bacterial cultures. Molecular techniques were utilized to identify the pure bacterial colonies.

### Isolation of genomic DNA and Amplification of 16S rRNA gene

The total bacterial genomic DNA was extracted from the bacterial cultures using the Quick-DNA™ Fungal/Bacterial Miniprep Kit (Zymo Research) following the manufacturer’s instructions. The 16S rRNA target gene was amplified by polymerase chain reaction (PCR) using the following, universal primers and enzymes: Forward primer (16S-27F: AGATTTGATCCTGGCT), reverse primer (16S-*1492R*: CGGTACCTTGTTGTTAC), and OneTaq® Quick-load® 2X Master Mix^32^. The PCR amplicons were run on an agarose gel and extracted with a Zymoclean™ Gel DNA Recovery Kit (Zymo Research). The extracted fragments were sequenced in the forward and reverse direction (Nimagen, BrilliantDye™ Terminator Cycle Sequencing Kit V3.1, BRD3-100/1000) and purified (Zymo Research, ZR-96 DNA Sequencing Clean-up Kit™). The purified fragments were analysed on an ABI 3500xl Genetic Analyzer (Applied Biosystems, ThermoFisher Scientific) for each reaction for every sample. The bacterial sequences were edited and aligned using MEGA X (version 10.2.6)^33^. The nBLAST database (http://www.ncbi.nlm.nih.gov/blast) was used to obtain closely matching sequences and to create consensus sequences for each bacterial isolate. Consensus sequences were then used to construct a phylogenetic tree using the Maximum-likelihood method in MEGA X. The Bootstrap values were generated from 1000 replicates. All the bacteria isolates' 16S rRNA nucleotide sequences were deposited at GenBank.

### Secondary metabolite production

The pure bacterial isolates were grown in liquid culture (broth) to induce the production of secondary metabolites, as described previously^34^. Briefly, a loop full of bacteria was inoculated into 50 mL of tryptic soy broth in Erlenmeyer flasks. These starter cultures were then incubated at 37 °C with constant shaking at 200 rpm for 7 days. After that, 5% of the starter culture was inoculated into a 1 L fermentation broth (tryptic soy broth) for large-scale fermentation. The cultures were then fermented at 37 °C with constant shaking at 90 rpm for 7 days. To obtain cell debris-free crude extracts, the cellular debris was collected by centrifugation at 4000 rpm for 10 min and filtered through a 0.2 nm nylon filter. The liquid mixtures containing bacterial secondary metabolites were then freeze-dried. The dried bacterial extracts were then suspended in a solvent, with a ratio of 100 ml of methanol to 10 g of dried bacterial culture^35^. The methanolic extracts were air-dried at room temperature under a constant stream of air and then stored at 4 °C until further analysis.

### Minimum inhibition concentration evaluation

The Mycobacterial strains: *M. smegmatis* mc^2^155, *M. aurum A*+ and *M. tuberculosis* H37Rv*,* were used in this study. *Mycobacterium smegmatis* mc^2^155 and *M. aurum A*+ were employed as surrogate strains in the preliminary screening for *M. tuberculosis* H37Rv. These two surrogate strains share some genome similarities with *M. tuberculosis* but are non-virulent. The microorganisms were stored in 50% glycerol at − 80 °C. Working stocks were cultured in vials containing Middlebrook 7H9 broth supplemented with 10% bovine albumin, catalase, dextrose, and sodium chloride (OADC-BBL/Becton–Dickinson, USA) and grown at 37 °C^36^. The bacterial crude extract stocks were prepared by dissolving the methanol crude extracts in 100% dimethyl sulfoxide (DMSO) and then diluted to 10% with water. The bacterial extracts' minimum inhibitory concentration (MIC) was then determined through the broth microdilution technique as described^35^. Briefly, *M. smegmatis* mc^2^155, *M. aurum A*+, and *M. tuberculosis* H37Rv were sub-cultured in Middlebrook 7H9 broth to reach an optical density (OD) of 0.2–0.3 at 600 nm. The crude extracts were pipetted into the first wells and then serially diluted to achieve final concentrations varied from 2500 to 19.53 µg/mL. Isoniazid was used as the positive control. Aliquots 100 µL inoculum of the test organism diluted 1:199 were pipetted into 96 well microtiter plates, and the plates were incubated at 37 °C for 72 h for *M. smegmatis* mc^2^155 and 144 h for *M. tuberculosis* H37Rv. Afterward, the results were evaluated using resazurin as a colorimetric indicator of mycobacterial growth. The MIC was defined as the lowest concentration capable of inhibiting bacterial growth, and all the assays were performed in technical and biological triplicates.

### LC-QTOF-MS/MS analysis

High-resolution mass spectra were obtained using an AB Sciex® X500R QTOF coupled to an AB Sciex® Exion LC system. Spectral data were obtained using information-dependent acquisition (IDA) at a mass range of 50–1500 Da. All methods, batches, and data were processed using OS Sciex® v3.1. The delustering potential was 80 V, the curtain gas (N2) was at 25 pounds per square inch (psi), the ion spray voltage was 5500 V, and the source temperature was 450 °C. Ion source gases 1 and 2 were at 45 and 55 psi, respectively. The collision energy was 10 eV for the MS scans and 20–50 eV for MS/MS scans. The IDA intensity threshold was 50 cycles per second. The aqueous mobile phase used was 1 mM ammonium formate in water, and the organic mobile phase was 0.5% formic acid dissolved in methanol. The gradient elution program for the organic mobile phase was set to start at 2% and end at 98% between 0 and 25 min, holding for 5 min before returning to 2% over 5 min to re-equilibrate for the next injection. The flow rate was 700 µL/min, and the run time was 35 min. A Kinetex® C18 column (5 µm, 100 Å, 150 mm × 6 mm) with a column protector was used. All solvents were sonicated for 10 min before use to remove bubbles.

#### Data processing and annotation

In order to classify the different types metabolite present in the three crude bacterial extracts, molecular networks were computed using the GNPS platform (https://ccms-ucsd.github.io/GNPSDocumentation/). Briefly, a molecular network was created with an MS/MS fragment ion tolerance of 0.025 Da^37^. The molecular network was then enriched with information from in-silico structure annotations from GNPS Library Search and variable Dereplicator using the GNPS MolNetEnhancer workflow (https://ccmsucsd.github.io/GNPSDocumentation/molnetenhancer/). The chemical class annotations were also performed using the ClassyFire chemical ontology^38^. Furthermore, the raw HPLC-qTOF data was converted to a “.abf” format by ABF converter software (http://www.reify.cs.com/AbfConverter) and then annotated using metabolic workflow on MS-DIAL software version 4.24^35,39^. The parameters for processing the files were as follows: mass range (MS1) m/z 50–1500; MS1 and MS2 tolerance of 0.01 and 0.025, respectively; [M + H] adducts ions with a peak height of 10,000. The tentative prediction of molecular formula and structure elucidation of the bacterial metabolites were processed using MS-FINDER software version 3.50 using the following parameters: MS1 and MS2 tolerances were set to 0.01 Da; formula calculation with isotopic ratio tolerance was set to 20% in-silico MS/MS fragmenter tree depth was set to 2; the databases selected were COCONUT(Natural product), UNDP(Natural product), ChEBI(Biomolecules), KNApSAcK(Natural product), PubChem(Biomolecules), and LipidMaps(Lipids). To reveal the differences in metabolic profiles of the bacterial crude extracts, a principal component analysis (PCA) plot was generated using Metaboanalyst 5.0 software (http://www.metaboanalyst.ca/)^39^. To expand the characterisation of the metabolomic potential of *B. subtilis*, S. *mycarofaciens*, and *B. licheniformis,* the Metaboanalyst 5.0 software (http://www.metaboanalyst.ca/) was further used to perform pathway enrichment as described by^40^. An over-representation analysis (ORA) was implemented using hypergeometric testing to determine whether certain metabolite sets were overrepresented compared to chance. Pathway topology analysis was conducted based on betweenness and out-of-degree centrality measures, evaluating the significance of each metabolite in each metabolic network. Potential targets were chosen using p-values from pathway enrichment analysis or impact values from pathway topology analysis, with an impact value threshold of 0.10 and a negative-log p-value threshold of 10. Altered pathways were identified, and potential functional analysis was carried out.

### Virtual screening of bacterial compounds

The potential mechanism of action of all tentatively identified bacterial compounds was evaluated using the virtual screening workflow in Schrödinger Release 2022-1. Briefly, the three-dimensional crystal structures of *M. tuberculosis* proteins (PDB:7Q52: PknG) and (PDB: 7VJT: Pks13) were downloaded from the Protein Data Bank (PDB). The raw crystal structures were prepared using the Protein Preparation Wizard (Schrödinger Release 2022-1), as described by Zong et al.^41^. Hydrogen atoms were added, the loop region was refined, H-bond assignments were optimised, and an OPLS-4 force field minimised energy. The co-crystallized heterogeneous ligands and water were removed, while polar hydrogens were added. The Receptor Grid Generator module generated the docking receptor grid configurations for all proteins using the coordinates of the previously co-crystallized ligands. The tentatively identified bacterial compounds were prepared by the LigPrep module (Schrödinger Release 2022-1) using the following parameters: energy minimised by an OPLS4 (Optimized Potentials for Liquid Simulations 4) force field, generated ionisation states at pH 7.0 + 2.0, and 32 multiple conformers per ligand. For Pks13, 3,8-bis(oxidanyl)-7-(piperidin-1-ylmethyl)-[1]benzofuro[3,2-c]chromen-6-one (7IJ) was used as control inhibitor while 2-azanyl-3-(4-fluorophenyl)carbonyl-indolizine-1-carboxamide (8ZC) was used as a control inhibitor for PknG. The Root Mean Square Deviation (RMSD) between the co-crystallized ligand and the ligand after docking was calculated to validate the docking protocol. A Virtual Screening Workflow (VSW) was used to screen a library of prepared compounds to obtain a hit list^42^. The QikProp module (Schrödinger Release 2022-1) was used to filter compounds. The remaining compounds were then screened based on Lipinski’s rule of five. Further, the returned compounds were subjected to three docking regimes of increasing precision using the Glide module^41^. Briefly, the bacterial compounds were docked against drug targets using a hierarchical approach that employed high-throughput virtual screening (HTVS), followed by standard precision (SP), and ultimately extra-precision (XP). The output hit from HTVS were filtered, and only 20% were selected for further SP docking. Similarly, from the SP docking outputs, only 20% were subjected to XP docking. Finally, 30% of the XP docking hits were retrieved and subjected to MM-GBSA free energy calculations. The workflow returned compounds that exhibit strong binding with an XP docking score below − 8 kcal/mol for each protein target. The Gaussian 16 Rev. C01 software was used for geometry optimisation and frequency calculations. The DFT calculations were performed using the M06-2X level of theory and 6-311++ G(*d,p*) basis set. The DFT computations were used to isolate the minimum energy conformation of a ligand along a potential energy surface. The compounds returned from the VSW and the modified compound were visualised in Gauss-view. The properties of the compounds were evaluated based on the $E$_HOMO_ and $E$_LUMO_. The equations used for the calculations are as follows:

1 $${\upmu } = { }\frac{{E_{LUMO} + E_{HOMO} }}{2}$$

2 $$\eta = { }\frac{{E_{LUMO} - E_{HOMO} }}{2}$$

3 $$\left( s \right) = \frac{1}{\eta }$$

### Molecular dynamics simulations

Molecular dynamics (MD) simulations in the study were performed by using the Desmond v5.3 module implemented in the Maestro interface (Schrödinger 2022‐1 suite). Molecular dynamics simulation systems were built by solvating the protein–ligand complexes with TIP4P explicit water molecules and placed in the centre of an orthorhombic box with boundary dimensions of (10 Å × 10 Å × 10 Å). The systems were neutralized by adding counter ions and a 0.15 M NaCl solution. The MD protocol involved minimisation, pre-production, and production MD steps. In the minimisation step, the system was allowed to relax for 2500 steps using the steepest descent approach. Then, the system's temperature was raised from 0 to 300 K with a small force constant on the protein to restrict any drastic changes. MD simulations were performed via NPT (constant number of atoms, constant pressure, i.e., 1.01325 bar and c, constant temperature, i.e., 300 K) ensemble. The Nose–Hoover chain method was used as the default thermostat with a 1.0 ps interval, and Martyna-Tobias-Klein as the default barostat with a 2.0 ps interval by applying an isotropic coupling style. Long-range electrostatic forces were calculated based on the Particle-mesh-based Ewald approach with the cut-off radius for columbic forces set to 9.0 Å. Finally, the system was subjected to production MD simulations for 200 ns for the free protein and protein–ligand complexes. During the simulation, the trajectories were written out every 1000 ps. The systems’ dynamic behaviour and structural changes were analysed by calculating the root mean square deviation (RMSD) and root mean square fluctuation (RMSF). Subsequently, the energy-minimized structure calculated from the equilibrated trajectory system was evaluated to investigate each ligand–protein complex interaction.

### Post molecular dynamic simulation analysis

The free energy change ($\Delta$G_*Bind*_) for the interaction between the receptor and the ligand to form a complex was computed using Molecular Mechanics Generalized Born Surface Area (MM/GBSA) as the summation of different interactions according to the equation below:

4 $$\Delta {\text{G}}_{Bind} = {\text{ E}}_{{{\text{Complex}}}} [{\text{E}}_{{{\text{Receptor}}}} {\text{E}}_{{{\text{Ligand}}}} ]$$

In the formula provided:

$\Delta$G_*Bind*_ represents the calculated relative free energy, which takes into account both ligand and receptor strain energy. E_Complex_ represents the MM-GBSA energy of the minimised complex, E_Ligand_ represents the MM-GBSA energy of the ligand after it has been removed from the complex and allowed to relax. E_Receptor_ represents the MM-GBSA energy of relaxed protein after separating it from the ligand^43^.

### Reaction-based in-silico modification of the selected strong binder compound

A virtual library generation approach was performed using the Enumeration module in Schrödinger. The identified strong binder for Pks13 datasets was selected as the molecular starting point for modification to expand the structural complexity and explore the chemical space using the Pathfinder (Schrödinger Release 2022-1) automated reaction-driven enumeration^44^. To achieve this, the compound was enumerated based on the 100 multiple synthetic routes and filtered based on the similarity to the active compound, SMiles ARbitrary Target Specification (SMARTS) removed compounds with reactive functional groups, and Pan assay Interfering Structures (PAINS) properties. Regardless of some novelty properties obtained from the new library comprising 1000 generated molecules, it is critical to extensively evaluate their molecular interaction with Pks13, respectively, as well as structural profiles^44^. In this regard, the library created was subjected to a VSW as described in “Virtual screening of bacterial compounds” section above. The strong binder returned from VSW was then subjected to MD simulation for 200 ns and binding free energy calculations described in sections “Molecular dynamics simulations” and “Post molecular dynamic simulation analysis” section.

### Ethical approval

Ethical approval for this study was approved by the Research Ethics Committee: Biological and Environmental Safety (REC: BES) of Stellenbosch University with reference number BEE-2022-3188.

## Results

### Identification of bacteria

In this study, 11 bacteria were isolated and identified from gold mine tailings. To identify the bacterial species, the 16 S rRNA sequences of strains were compared to the GenBank sequence database using BLASTn. The bacterial strains that showed high quality sequences were used to construct a phylogenetic tree using the maximum likelihood approach in MEGA X. The relationships among the bacteria species are depicted in Fig. S3. The bacterial sequences were submitted to GenBank and assigned accession numbers (OM182829-OM182840), as listed in Table 1. The study investigated the secondary metabolite diversity and antimycobacterial activity of eleven identified bacteria, belonging to the genera *Bacillus* (six), *Micrococcus* (two), *Streptomyces* (one), *Staphylococcus* (one), and *Kocuria* (one). The crude bioactive extracts of the bacterial strains were tested for their antimycobacterial efficacy and screened in-silico for better-targeted anti-*Mycobacterium* validation. A minimum inhibitory concentration (MIC) below 2.5 mg/mL was considered an indicator of inhibitory activity. The results showed that three bacterial isolates, namely *Bacillus subtilis*, *Bacillus licheniformis*, and *Streptomyces mycarofaciens*, exhibited significant activity against three strains of Mycobacterium, including M. smegmatis mc^2^155, *M. aurum* A+, and *M. tuberculosis* H37Rv. The crude extracts from B. subtilis showed the most potent efficacy against all three strains, with MIC values ranging from 0.3125–0.625 mg/mL. The crude extracts from B. licheniformis strongly inhibited all three test strains, with MIC values ranging from 1.25 to 2.6 mg/mL. The crude extracts from *S. mycarofaciens* also exhibited strong inhibition against *M. smegmatis* mc^2^155 and *M. aurum* A+, with MIC values ranging from 0.325 to 2.25 mg/mL.

**Table 1:** Minimum inhibition concentration of bacterial crude extracts against *M. smegmatis* mc^2^155, *M. aurum A*^+^ and *M. tuberculosis* H37Rv expressed in mg/mL.

Sample ID	Predicted identity	Accession number	MIC (mg/mL)		
			*M. smegmatis* mc^2^155	*M. aurum A*^+^	*M. tuberculosis* H37Rv
KN1	*Micrococcus luteus*	OM182829	> 2.5	> 2.5	> 2.5
KN2	***Streptomyces mycarofaciens***	**OM182830**	**2.5–1.25**	**2.5–0.325**	> 2.5
KN3	*Bacillus simplex*	OM182831	> 2.5	> 2.5	> 2.5
KN4	*Bacillus *sp.	OM182832	> 2.5	> 2.5	> 2.5
KN5	*Micrococcus luteus*	OM182833	> 2.5	> 2.5	> 2.5
KN6	*Staphylococcus saprophyticus*	OM182834	> 2.5	> 2.5	> 2.5
**KN7**	***Bacillus licheniformis***	**OM182835**	**2.5–0.3125**	**2.5–0.625**	**2.5–1.25**
KN8	*Kocuria rhizophila*	OM182836	> 2.5	> 2.5	> 2.5
KN10	*Bacillus paralicheniformis*	OM182838	2.5	> 2.5	> 2.5
KN11	*Bacillus mobilis*	OM182839	> 2.5	> 2.5	> 2.5
**KN12**	***Bacillus subtilis***	**OM182840**	**0.1625**	**0.3125**	**0.625–0.3125**
**Control**	**Isoniazid**		**0.125–0.0625**	**0.125–0.0625**	**0.125–0.0625**

*Isoniazid was used as a positive control for antimycobacterial activity.

^**†**^MIC stands for minimum inhibitory concentration.

### Metabolite profiling of bacterial crude extract

**Figure 1:**
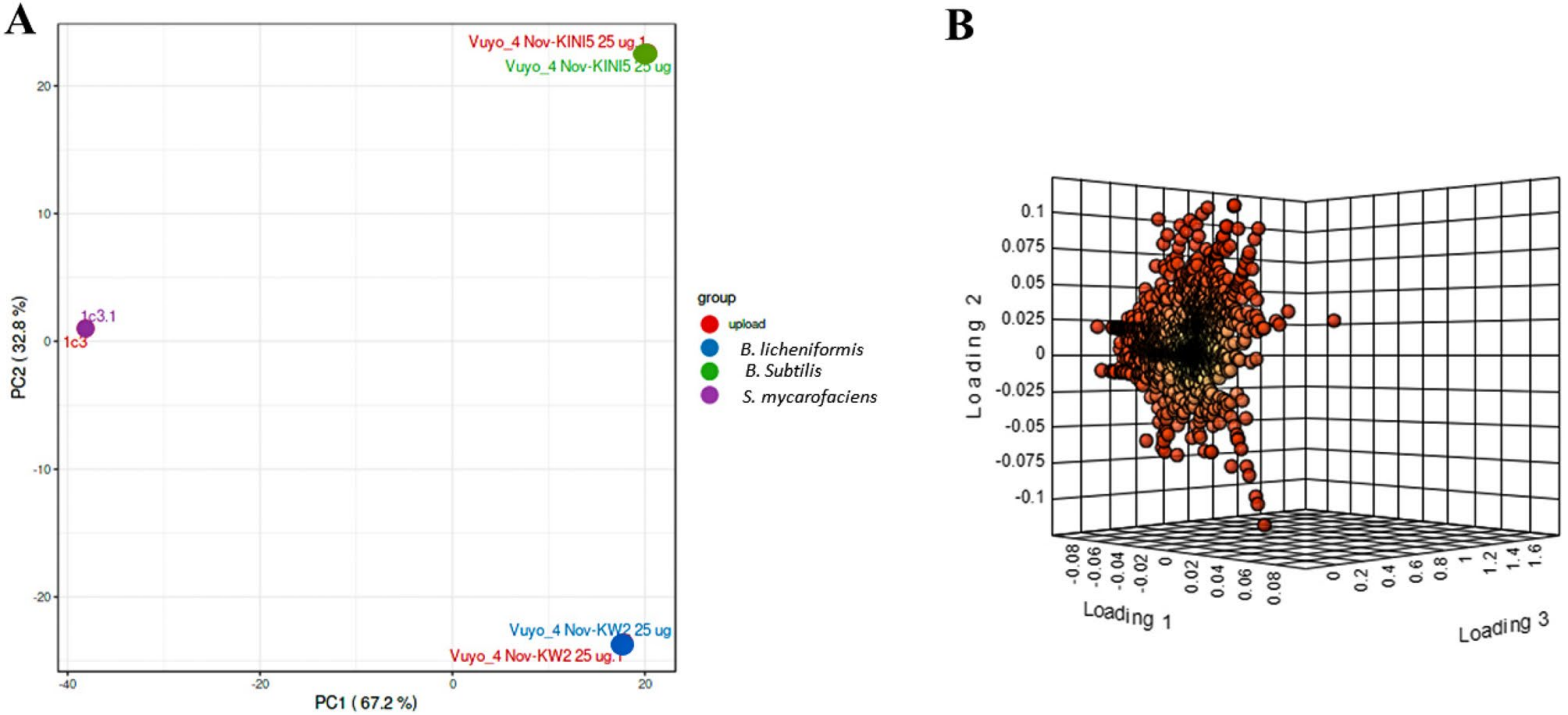
Principal component analysis (PCA) of metabolite data acquired by HPLC-qTOF of three bacterial crude extracts in positive ionisation mode. (**A**) PCA scores plot comparing metabolites present in crude extracts from *B. subtilis*, *B. licheniformis,* and *S. mycarofaciens.* (**B**) Loading plot from PCA analysis.

**Figure 2:**
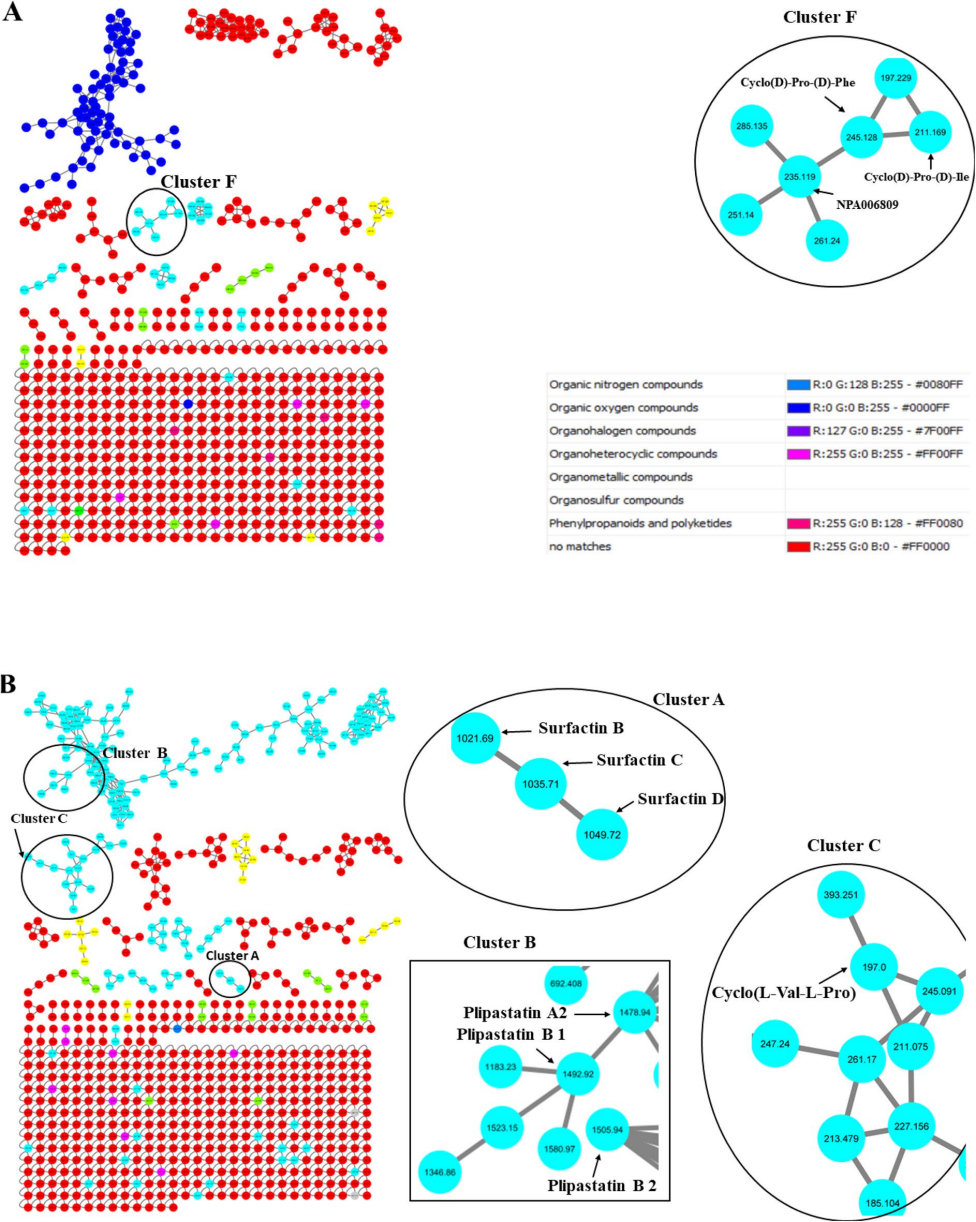

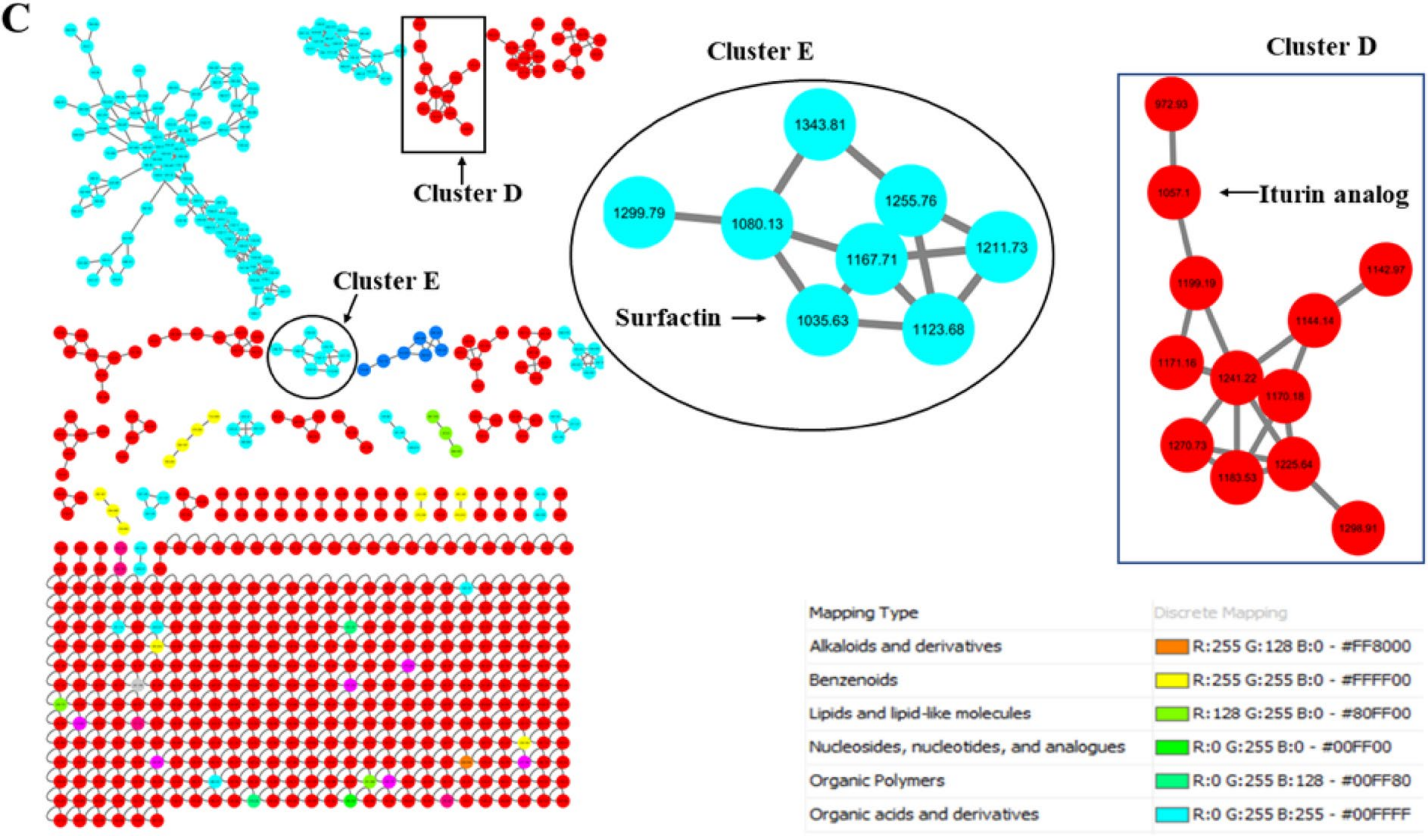
Molecular networking of bacterial crude extracts of (**A**) *S. mycarofaciens* (**B**) *B. subtilis* and (**C**) *B. licheniformis.* Unknown compounds in red are not characterised. The molecular network annotation indicates that novel compounds are abundant in *S. mycarofaciens*, *B. subtilis*, and *B. licheniformis.* Molecular networking of bacterial crude extracts of (**A**) *S. mycarofaciens* (**B**) *B. subtilis* and (**C**) *B. licheniformis.* Unknown compounds in red are not characterised. The molecular network annotation indicates that novel compounds are abundant in *S. mycarofaciens*, *B. subtilis*, and *B. licheniformis.*

There is limited information on the effectiveness of functional crude extracts from bacteria found in South African gold mine tailings against mycobacteria. To better understand the metabolic potential of these bacteria and their influence on antimycobacterial activity, a comprehensive untargeted metabolomics analysis was conducted on the crude extracts (Fig. S5). A principal component analysis (PCA) approach was constructed from the HPLC-qTOF data of the three active crude extracts against the mycobacteria species. The secondary metabolites produced by the three bacteria were separated and grouped into three clusters, corresponding to *B. subtilis*, *B. licheniformis*, and *S. mycarofaciens,* as depicted in Fig. 1A. The three bacteria strains showed a similar pattern in the potential secondary metabolites identified, with the differences mainly due to the presence of organic nitrogen and oxygen compounds. This was demonstrated by the clustering in the loadings and molecular network as seen in Figs. 1B and 2.

In the current study, molecular networking was performed to identify metabolites tentatively. GNPS computed the network based on spectral similarities, which grouped the metabolites into clusters of different nodes. The clusters are depicted in Fig. 2. The compounds from *S. mycarofaciens*, *B. subtilis*, and *B. licheniformis* belong to various classes of natural compounds, with a high quantitative variation in the pool of compounds shown in Fig. 2 and Table S1, which is strongly correlated with antimicrobial activity. Cyclic peptides with antimicrobial potential were putatively identified from *S. mycarofaciens* and are shown in cluster F at m/z 245.128, 211.169, and 235.119, as depicted in Fig. 2A. Three classes of tentatively identified compounds that might have contributed to the antimycobacterial activity were found to have molecular weights similar to cyclic lipopeptides produced by *Bacillus* sp. These included plipastatin and surfactin. In cluster A, the nodes for various surfactin isomers were directly connected, as depicted in Fig. 2. In cluster B, a direct relationship was observed between plipastatin B 1 at m/z 1492.92 and Plipastatin A 2 at m/z 1478.94, with an additional node of another plipastatin analogue observed at *m/z* 1506.94. Cluster C featured a node for cyclo(L-Val-L-Pro) at *m/z* 197.0, which was also associated with a possible cyclic-peptide compound at m/z 261.17 (Fig. 2). In cluster D, shown in Fig. 2C, the node at *m/z* 1057.1 was classified as an unknown compound, but it has an m/z that is associated with iturin analogue (1057). In cluster E, the node with *m/z* 1035.63 corresponding to surfactin (Fig. 2C). Most of the metabolites present in the bacterial crude extracts have not been fully characterised and remain unknown, as depicted in Fig. 2. The tentatively identified metabolites from the three bacterial crude extracts belong to aminocyclitol glycosides, alpha-amino acids, and derivatives; cyclic depsipeptides, phenylpropanoids, and polyketides; benzenoids, organic acids, and alpha-amino derivatives; organic heterocyclic compounds, and organic oxygen compounds (Table S1). GNPS did not assign structure to the respective nodes. Thus, MS-FINDER was used to predict the molecular formulas (Table S1).

### Characterization and functional analysis of the key metabolic pathways

**Figure 3:**
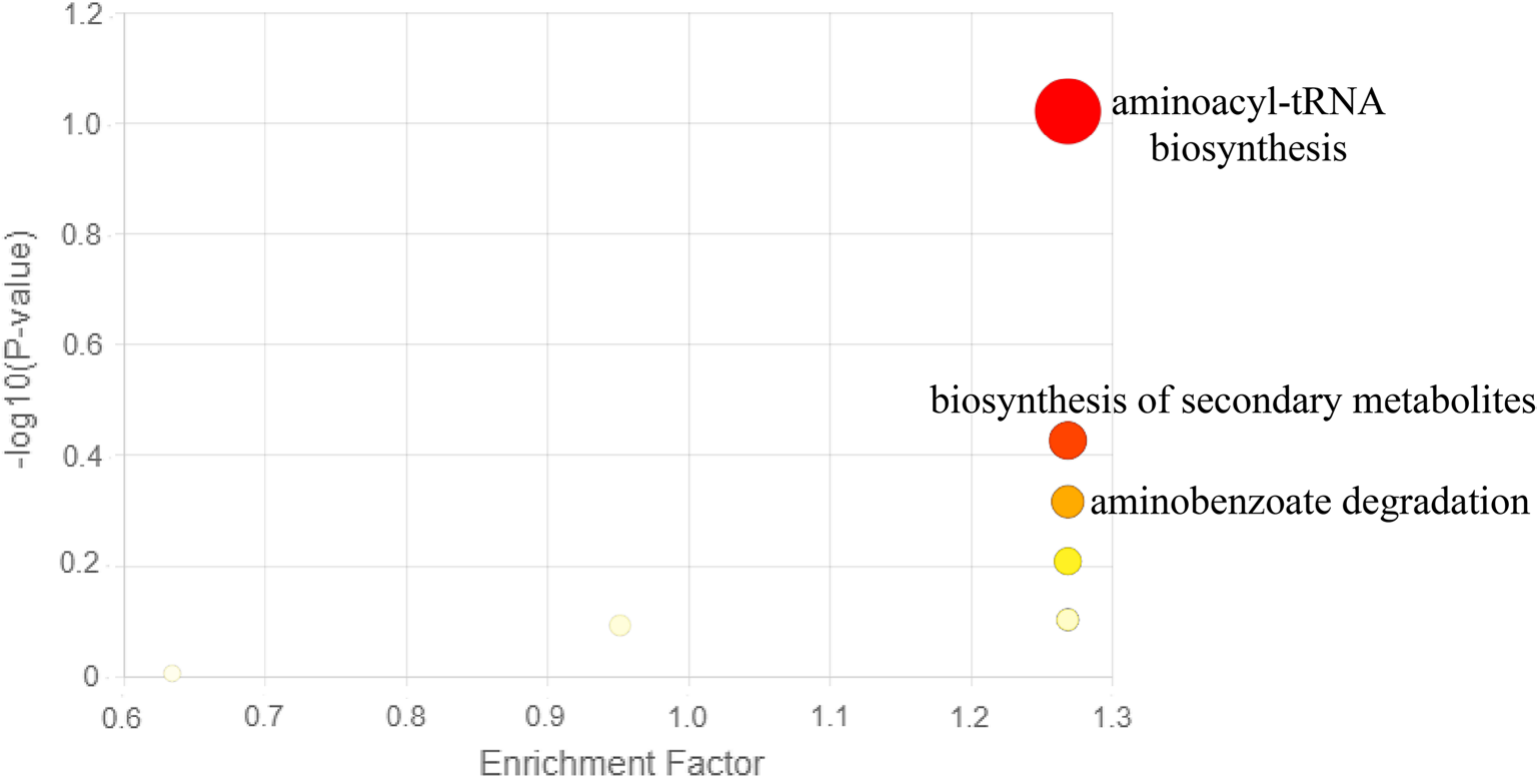
Metabolic pathway analysis generated with the MetaboAnalyst based on the metabolites identified from *B. subtilis*, *S. mycarofaciens*, and *B. licheniformis* crude extracts*.* The pathway enrichment analysis was based on the p-values on the Y-axis to determine the significance of the metabolites. The range of colours on the plot, ranging from yellow to red, represents the varying levels of significance of the metabolites for the enrichment analysis.

An investigation was conducted on the metabolic pathways of *B. subtilis*, *S. mycarofaciens,* and *B. licheniformis,* which exhibited the strong antimycobacterial activity. Metaboanalyst was used to process the data. The pathways enrichment analysis revealed some interesting pathways such as the biosynthesis of secondary metabolites that have antimicrobial potential, aminoacyl-tRNA biosynthesis, novobiocin biosynthesis, and aminobenzoate degradation (Fig. 3; Supplementary file 2).

### Virtual screening and binding dynamics analysis

**Figure 4:**
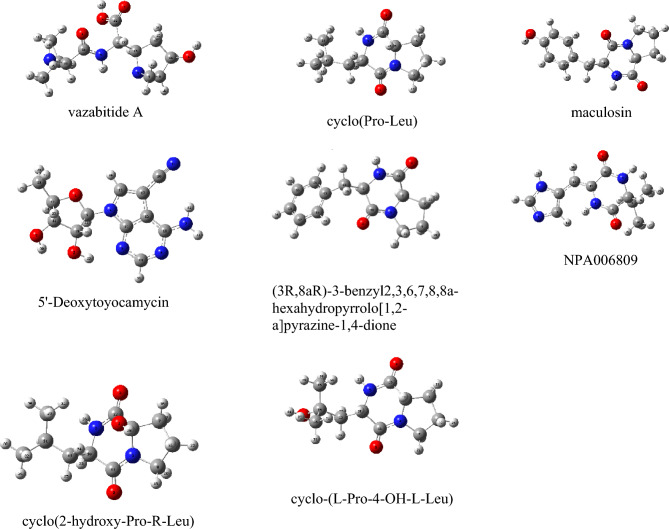
DFT optimised geometric structures of the identified compounds returned from the virtual screening workflow.

The RMSD values of the co-crystalized and re-docked ligands of PknG and Pks13 were 0.61 and 1.19, respectively, as shown in Fig. S4. The bacterial compounds that were tentatively identified were subjected to VSW to determine the strongest binding postures and explore their mode of interaction. Based on the output of Glide-XP docking, eight compounds exhibited strong XP docking profiles to the two target proteins, with a range of − 8.8 kcal/mol to − 11.9 kcal/mol. The identified bacterial compounds' optimized geometries were obtained through DFT calculations, as depicted in Fig. 4. A frequency analysis was conducted to determine the stability of the optimized scaffolds. The analysis confirmed that the scaffolds represent the lowest energy states, and no negative frequencies were obtained. Furthermore, the docking scores of the quantum mechanics optimised scaffolds were compared with those obtained from ligands optimized using the OPLS4 force field. The results showed that the docking scores of both quantum mechanics optimized and OPLS4 force field optimized are similar, which increases the confidence of the docking protocol.

**Figure 5:**
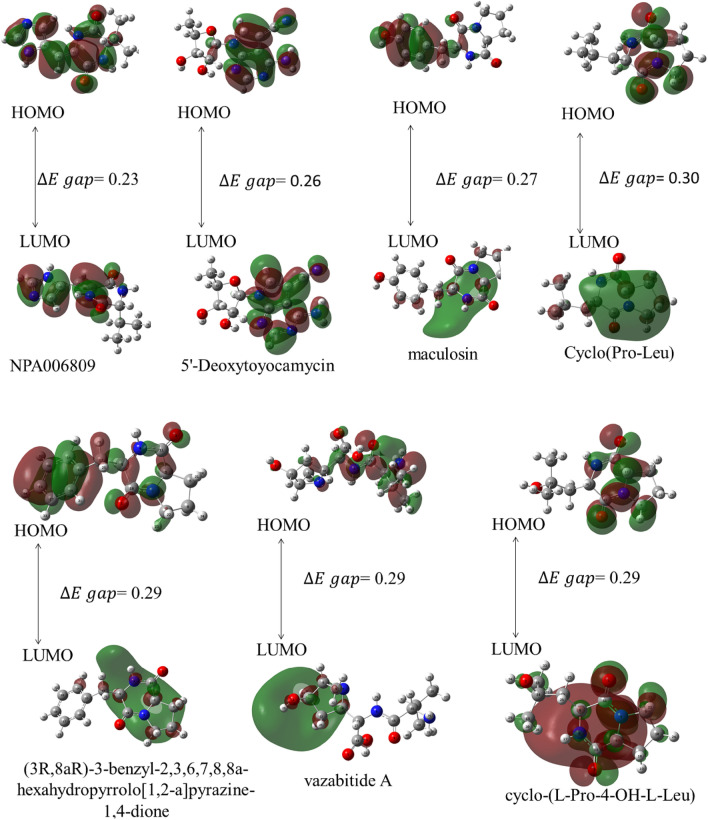
Depiction of the HOMO–LUMO surface maps computed with M06-2X/6-311++ (*d,p*). $\Delta {\text{E}}$ is measured in eV. Depiction of the HOMO–LUMO surface maps computed with M06-2X/6-311++ (*d,p*). $\Delta {\text{E}}$ is measured in eV.

The study computed the reactivity of the compounds using the M06-2X level of theory and 6-311++G (d,p) basis set. The chemical potential (µ), chemical hardness (ɲ), chemical softness (s), electronegativity (χ), and electrophilic index (ω) global reactivity descriptors of the compounds were calculated and can be found in Table S2. Notably, the lowest unoccupied molecular orbital (LUMO) is expected to accept electrons, while the highest occupied molecular orbital (HOMO) is an electron donor. The difference between the HOMO and LUMO yields an energy gap ($\Delta E$). The ionization energy is computed by minus LUMO. A lower energy gap ($\Delta E$) indicates higher reactivity and is associated with a soft compound. Conversely, a higher energy gap ($\Delta E$) implies greater stability and lower reactivity. In this study, the energy gap ($\Delta E$) ranged from 0.23 to 0.31 eV, indicating only a small difference. The compound NPA006809 exhibited the smallest energy gap of 0.23 eV and the lowest ionisation energy (0.27 eV). These results indicate that the compounds are soft and can interact as electron acceptors (Table S2 and Fig. 5). The high docking scores also support that the compounds are reactive as shown in Table 2. Additionally, the LUMO surface was localized under regions containing nitrogen rings.

**Table 2:** Virtual screening of bacterial compounds against *M. tuberculosis* macromolecular targets (PknG and Pks13).

Complex	Mol MW (170–725)	Dipole (1.0–12.5)	SASA	*QplogS (− 6.5 to 0.5)	PSA (7.0–200.0)	Volume	%Human oral absorption	XP GScore^a^ (kcal/mol)	XP Gscore^b^	Pre-MD-MM-GBSA^a^ ΔG_*bind*_ (kcal/mol)	Pre-MD-MM-GBSA^b^ ΔG_*bind*_ (kcal/mol)
PknG-ligand complex											
Maculosin	260.29	2.96	528.57	− 1.09	92.29	875.01	59.48	− 9.4	− 9.4	− 35.7	− 35.7
NPA006809	234.26	3.85	466.23	− 2.38	103.50	775.73	71.17	− 9.4	− 9.4	− 39.8	− 39.8
5′-Deoxytoyocamycin	275.27	6.02	487.25	− 3.16	128.19	829.80	54.70	− 9.3	− 9.3	− 38.0	− 38.2
(3R,8aR)-3-benzyl-2,3,6,7,8,8a-hexahydropyrrolo[1,2-a]pyrazine-1,4-dione	244.29	1.80	516.11	− 1.20	69.75	852.12	72.70	− 9.1	− 9.0	− 41.1	− 41.0
Cyclo(Pro-Leu)	210.28	1.90	458.39	− 0.05	69.87	764.78	68.98	− 8.9	− 8.9	− 42.6	− 42.6
Cyclo-(L-Pro-4-OH-L-Leu)	226.28	3.50	473.85	− 0.50	88.06	789.50	64.63	− 8.8	− 8.8	− 42.8	− 42.8
Cyclo(2-hydroxy-Pro-R-Leu)	226.26	2.14	330.80	0.11	87.68	781.66	61.59	− 8.8	− 8.5	− 34.7	− 35.1
Control (8ZC)								− 9.5	− 9.5	− 48.4	− 48.4
Pks13-ligand complex											
Vazabitide A	271.32	13.40	515.62	− 0.90	133.66		27.76	− 11.9	− 8.218	− 47.6	− 47.6
Control (7IJ)								− 8.2	− 8.2	− 42.0	− 42.0

^a^Ligands docked using OPLS4 force field.

^b^Ligands docked after M06-2X/6-311++ G(*d,p*) optimization. SASA (solvent accessible surface area). MW (molecular weight).

**Figure 6:**
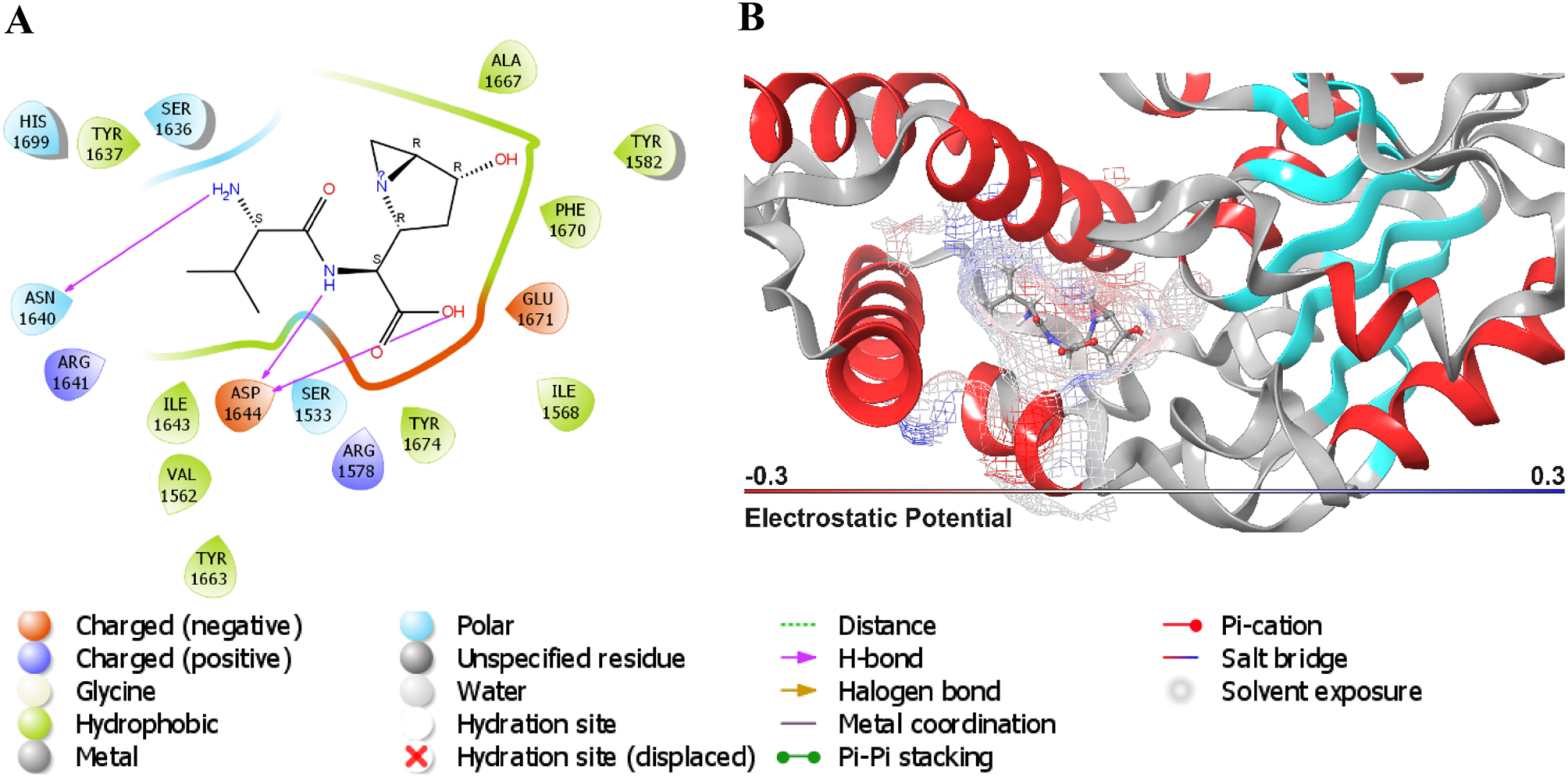
A concise overview of the interaction of vazabitide A with Pks13. (**A**). 2D representation (**B**). 3D representation.

Notably, vazabitide A exhibited the strongest XP docking score of − 11.9 kcal/mol against Pks13 and a ΔG_bind_ of − 47.6 kcal/mol, as shown in Table 2. However, the percentage human oral absorption of vazabitide A was calculated to be relatively low at 27.76%. The interaction between vazabitide A and Pks13 was visualized, and it was found that the compound was anchored in the hydrophobic pocket of Pks13. The compound interacted with proximal amino acid residues through hydrogen bonding with Asn1640, and Asp1644, had a positive charge interaction with Arg1578 and Arg164, and had polar interactions with Ser1533, Asn1640, Ser1636, and His169 as depicted in Fig. 6. These interactions collectively contributed to the observed Δ*G*_bind_ − 47.6 kcal/mol (Table 2). The control (7IJ) ligand exhibited a docking score of − 8.2 kcal/mol and an MM-GBSA value of − 42.0 kcal/mol. The interaction with the nitrogen rings was as expected, as per HOMO–LUMO results.

Seven compounds, including maculosin, NPA006809, 5′-Deoxytoyocamycin, (3R,8aR)-3-benzyl-2,3,6,7,8,8a-hexahydropyrrolo[1,2-a]pyrazine-1,4-dione, cyclo(Pro-Leu), cyclo-(L-Pro-4-OH-L-Leu), and cyclo(2-hydroxy-Pro-R-Leu) exhibited strong XP docking scores with PknG as shown in Table 2. Among them, cyclo-(L-Pro-4-OH-L-Leu) had the highest affinity of − 42.8 kcal/mol with a pre-MD-MM-GBSA ΔG_*bind*_ value. Maculosin and NPA006809 exhibited an XP docking score of − 9.1 kcal/mol with PknG, as shown in Table 2, are depicted in Fig. S6.

**Figure 7:**
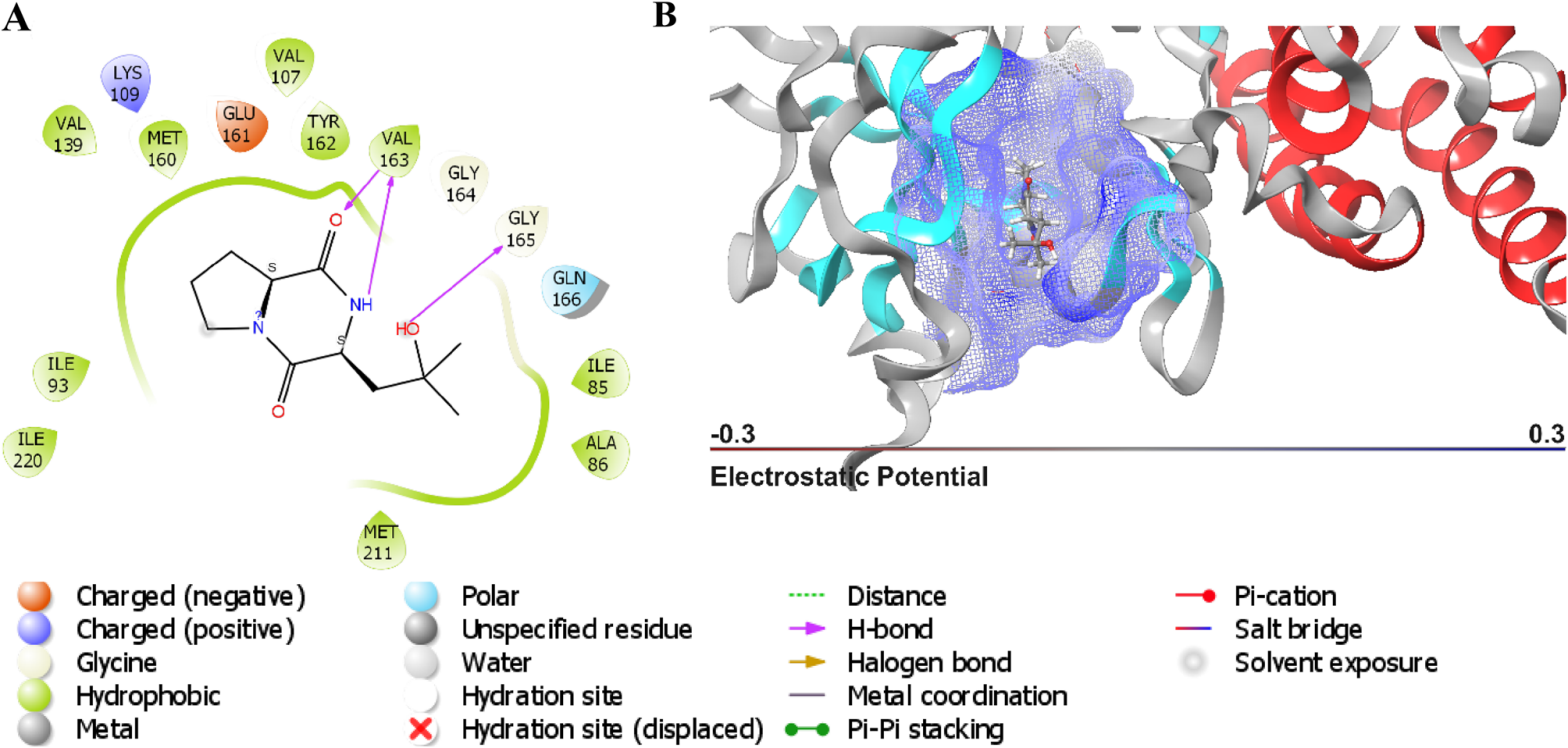
A concise superposition of the interaction of cyclo-(L-Pro-4-OH-L-Leu) with the binding pocket of PknG in two dimensions. (**A**) 2D representation (**B**) 3D representation.

The hydrophobic interaction of cyclo-(L-Pro-4-OH-L-Leu) in the active site of PknG was among the contributing attractive forces to its high XP docking score. Additionally, cyclo-(L-Pro-4-OH-L-Leu) displayed multiple types of interactions with proximal amino acid residues, including hydrogen bonding with Val163 and Gly165, polar interactions with Gln166, negative charge interaction with Glu16, and positive interaction with Lys109 (Fig. 7). The complex was visualized in both two and three dimensions to gain further knowledge of the interactions that contributed to the highest ΔG_*bind*_ observed on PknG-cyclo-(L-Pro-4-OH-L-Leu). The control ligand had an XP docking of − 9.5 kcal/mol and an MM-GBSA value of − 8.4 kcal/mol. To gain more comprehensive insights into the binding dynamics of the PknG-cyclo-(L-Pro-4-OH-L-Leu) and Pks13-vazabitide A complexes, MD simulations and post-MD-ΔG_*bind*_ simulations were computed.

Based on the pre-MD binding energies calculated using VSW, two ligands (6 and 8) were chosen for further analysis through MD simulations. The simulations were conducted for 200 ns for both the protein–ligand complexes and the protein alone, and the obtained trajectories were analysed to gain a better understanding of the interaction dynamics of these complexes. Additionally, the co-crystallized ligands were also simulated for comparison.

**Figure 8:**
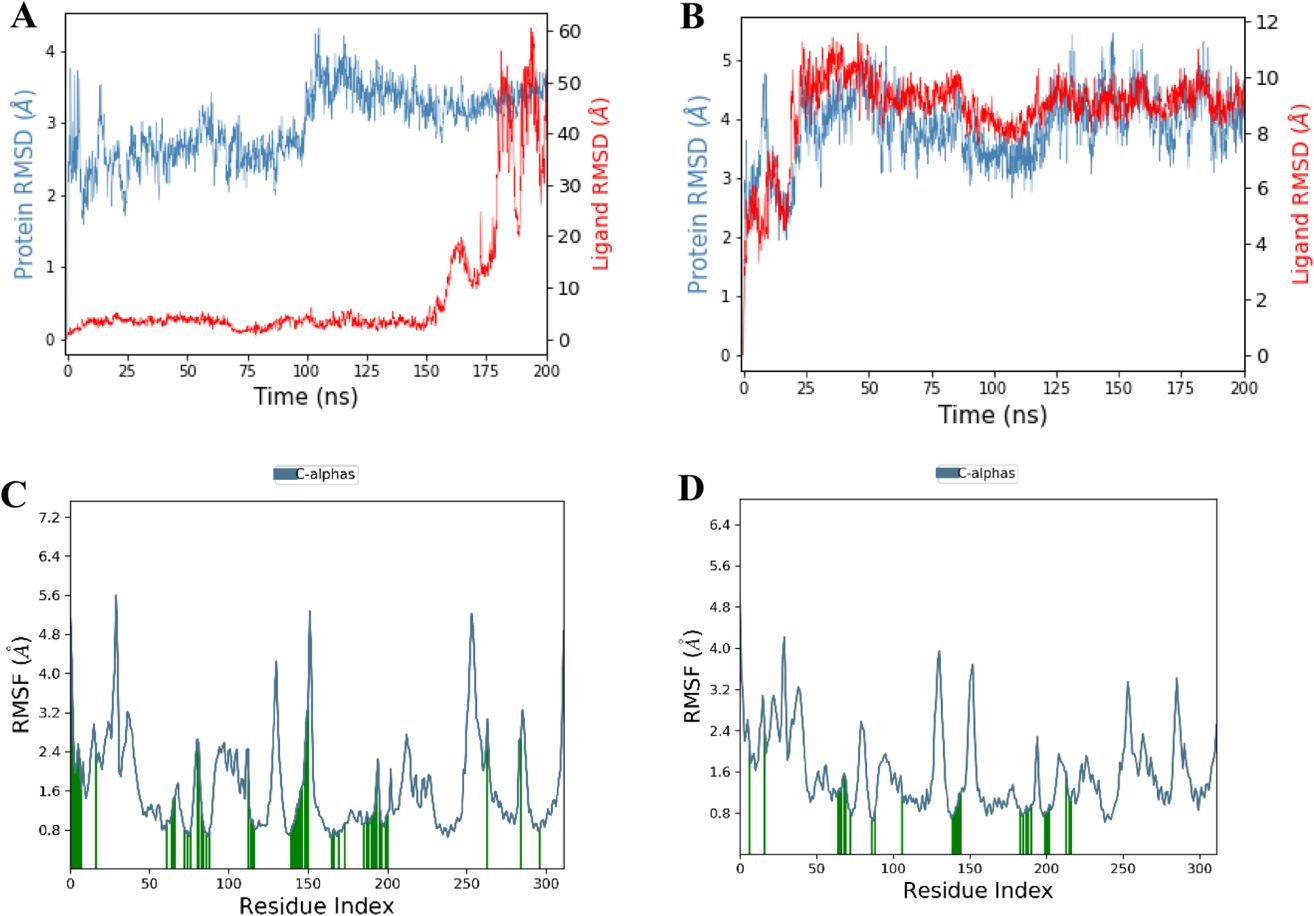
(**A**) RMSD of PknG Cα-atoms and Cyclo-(L-Pro-4-OH-L-Leu) over a 200 ns simulation. (**B**) RMSD for PknG Cα-atoms and the co-crystallized ligand (**8ZC**) over a 200 ns simulation. (**C**) RMSF per residue of PknG in complex with Cyclo-(L-Pro-4-OH-L-Leu). (**D**) RMSF per residue of PknG in complex with 8ZC ligand.

The MD simulations conducted on the unbound PknG protein showed a gradual increase in RMSD from 1.6 to 2.4 Å over 50 ns then stabilized for the remaining simulation period, indicating the protein's stability as shown in Fig. S1. On the other hand, the RMSD of Pks13 increased from 1.2 Å during the initial 25 ns and then stabilized at approximately 2.8 Å for the remaining simulation period as depicted in Fig. S2. During the 200 ns MD simulation of the PknG complexed with cyclo-(L-Pro-4-OH-L-Leu), the RMSD of PknG Cα-atoms remained relatively stable, fluctuating in the range 3.0–1.5 Å for the first 100 ns. However, after 100 ns, the RMSD increased slightly to 3.5 Å and remained constant for the rest of the simulation period (Fig. 8A). The RMSD of cyclo-(L-Pro-4-OH-L-Leu) remained stable mostly stable except for a sudden spike fluctuation at 150 ns, in which the ligand slightly shifted from the designated active site and started interacting with the protein’s loop (Fig. 8A). The stability of the PknG-co-crystallized ligand (8ZC) complex was investigated during a 200 ns MD simulation, and the RMSD of PknG Cα-atoms ranged from 5.6 to 3.2 Å (Fig. 8B), indicating that the complex was not stable during the simulation period. The RMSD of 8ZC ranged around 9 Å, indicating that the ligand is not a strong binder of PknG. A comparison of the RMSD values of PknG-cyclo-(L-Pro-4-OH-L-Leu) and the PknG-8ZC revealed that Cyclo-(L-Pro-4-OH-L-Leu) had a more favourable binding mode with PknG for most parts of the simulation. However, the RMSD profiles of both ligands show that the ligands are not strong binders of PknG. The RMSF revealed that residues 43–51, 147–152, 171–173, and 272–277 were relatively mobile, with RMSF values above 3.0, in both the PknG-cyclo-(L-Pro-4-OH-L-Leu) complex and PknG-8ZC complexes as shown in Fig. 8C,D. The RMSD value of PknG, when it was complexed with maculosin, was around 4 Å, indicating that the complex formed was not stable. Similarly, the RMSD value of PknG when it was complexed with NPA006809 was approximately 6 Å, which also suggests that the complex formed in this case was not stable. These results have been illustrated in Fig. S7.

**Figure 9:**
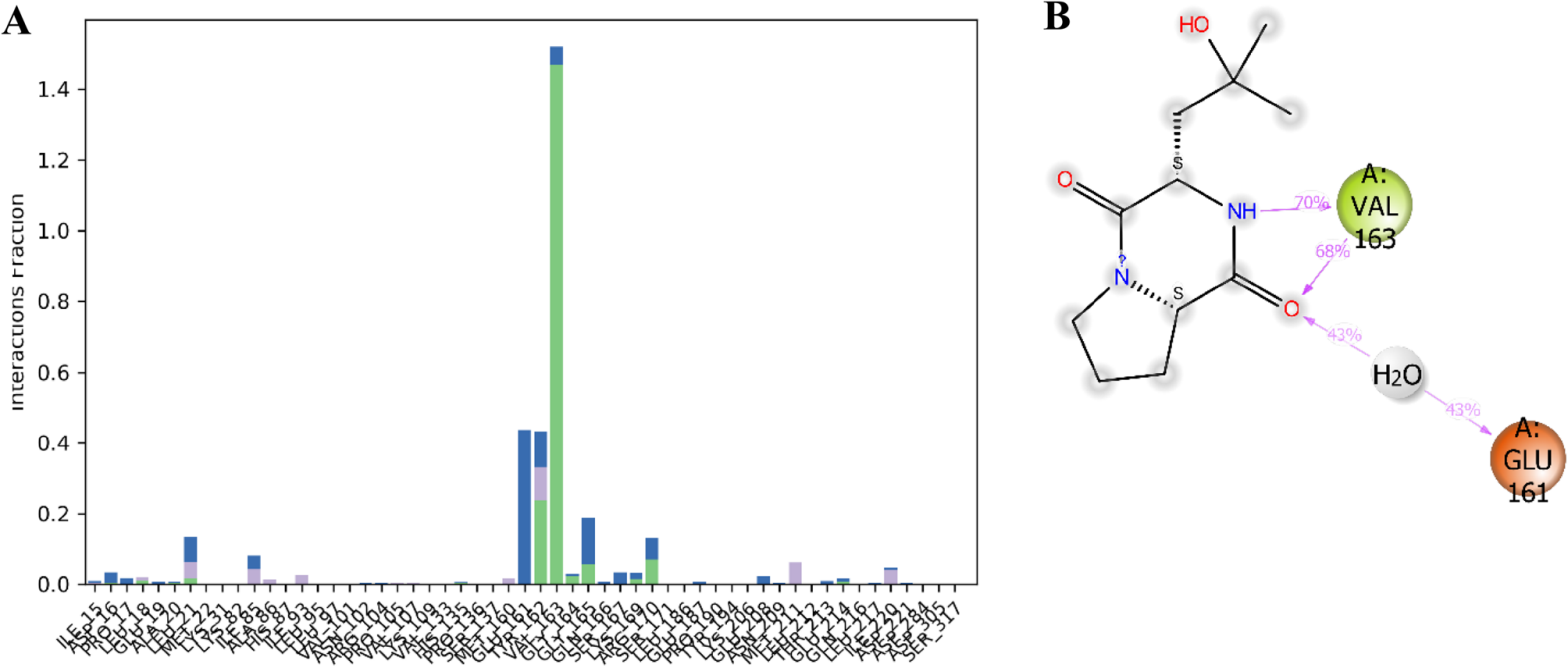
Interaction Fraction summary of PknG-cyclo-(L-Pro-4-OH-L-Leu) contacts. This graph is normalised by the total simulation time. (**A**) Interaction fraction of PknG with the ligand cyclo-(L-Pro-4-OH-L-Leu) (**B**) Interactions occurred for more than 30% of the 200 ns MD simulation.

During the 200 ns simulation, the PknG complex with cyclo-(L-Pro-4-OH-L-Leu) remained partially stable, mostly due to non-covalent interactions. These included hydrogen bonds involving Lue18, Leu21, H315, Val162, Val163, Gly164, Gly165, Lys169, Arg170, and Glu214, as well as hydrophobic interactions involving Lue21, Ile85, Ala86, Ile93, Met160, Tyr162, Met211, and Ile220. Water bridges were also observed, involving Ile15, Asp16, Pro17, Glu19, ALA20, Leu21, Ile85, Asn102, Arg104, Glu161, Tyr162, Val163, Gly164, Gly165, Glu166, Ser167, Lys169, Arg170, Glu187, Glu208, Thr213, Glu214, and Ile220, along with ionic interactions, as illustrated in Fig. 9A. During the simulation, the ligand's carbonyl group at position 3 interacted with protein residues through water bridges for 43% of the 200 ns MD simulation time with Glu161, and through hydrogen bonding with Val163 for 63% of the 200 ns MD simulation time. Additionally, the ligand's amide group at position 4 formed a hydrogen bond with the protein residue Val163, as illustrated in Fig. 9B.

**Figure 10:**
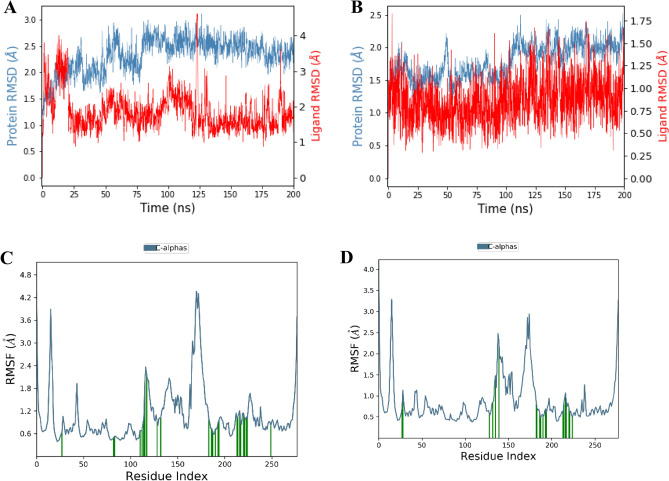
(**A**) RMSD of Pks13 Cα-atoms and the vazabitide A over a 200 ns simulation. (**B**) RMSD for Pks13 Cα-atoms and 7IJ over a 200 ns simulation. (**C**) RMSF per residue of Pks13 in complex with the vazabitide A. (**D**) RMSF per residue of Pks13 in complex with 7IJ.

The Root Mean Square Deviation (RMSD) of Pks13 Cα-atoms complexed with vazabitide A increased gradually from 1.5 to 2.4 Å during the first 50 ns and then remained stable between 2.1 and 2.4 Å for the next 200 ns, as shown in Fig. 10A. The low RMSD value, which remained below 3 Å, indicates that the complex was stable. In contrast, the co-crystallized ligand (7IJ) also formed an excellent stable complex with the RMSD averaging around 1.5 Å, as shown in Fig. 10B. The RMSF of Pks13 Cα-atoms in both systems showed that the residues did not fluctuate significantly, except for the mobile residues (10–20 and 170–180) as shown in Fig. 10C,D.

**Figure 11:**
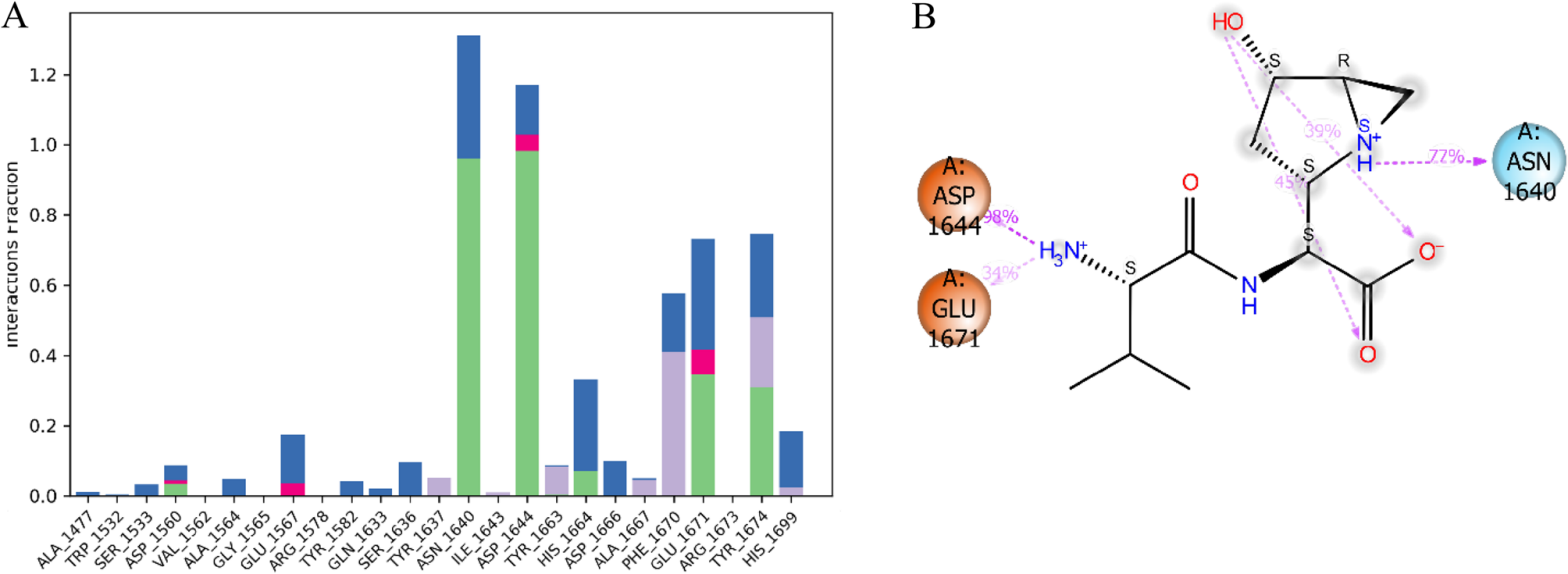
Interaction Fraction summary of Pks13-vazabitide A contacts. This graph is normalised by the total simulation time. (**A**) Histogram of the interaction fraction of the vazabitide A with Pks13. (**B**) Interactions that occurred for more than 30 $\%$ of the 200 ns MD simulation.

During the study, it was observed that the stability of the Pks13-vazabitide A complex was significantly influenced by non-covalent interactions. These interactions included hydrogen bonding with key protein residues such as Asp1562, Asn1640, Asp1644, His1664, Glu1671, and Tyr1674. Additionally, water bridges also played a critical role in contributing to the stability of the complex, with residues such as Ala1477, Ser1533, Asp1560, Ala1564, Glu1567, Try1582, Gln1633, Ser1636, Asn1640, Asp1644, His1664, Asp1666, Ale1667, Phe1670, Glu1671, Try1674, and His1699 being involved. Ionic interactions also enhanced stability, with residues such as Asp1560, Glu1567, Asp1644, and Glu167 being involved in the process, as presented in Fig. 11A. Figure 11B shows that during the 200 ns MD simulation time, the amino group of vazabitide A located at position 7 formed a hydrogen bond with Glu1671 and Asp1644, staying engaged in electrostatic interactions with the two protein residues for 34% and 98% of the simulation time, respectively. Additionally, the amide group located at position 5 of vazabitide A formed a hydrogen bond with Asn1640 for 77% of the 200 ns MD simulation time. Vazabitide A was also observed to form intramolecular hydrogen bonds between the hydroxyl group at position one and a carboxylic acid group at position 14.

Table 3 presents the results of using post-MD MM-GBSA binding free energy to determine and compare the binding affinities of selected ligands against co-crystallized ligands as control. Cyclo-(L-Pro-4-OH-L-Leu) showed a binding free energy of − 21.1 kcal/mol for PknG, while 8ZC, the control ligand, showed a binding free energy of − 31.2 kcal/mol. The co-crystallized ligand (7IJ) showed a more negative binding free energy of − 83.4 kcal/mol for Pks13, compared to vazabitide A, with a binding free energy of − 7.2 kcal/mol. The pre-MD MM-GBSA values of vazabitide A and cyclo-(L-Pro-4-OH-L-Leu) to Pks13 and PknG were lower than post-MD MM-GBSA values. This is because, in molecular docking, the protein is rigid and doesn't account for all the entropic and enthalpic factors that play a role in the formation of the protein–ligand complex. The ΔG_*bind*_ vdW interactions contributed significantly to the stability of the Pks13-vazabitide A complex. Table 3 shows that ΔG_*bind*_*Lipophilicity*, ΔG_*bind*_*Solvation* GB, ΔG_*bind*_*Hbond*, and ΔG_*bind*_*Coulomb* are some of the interactions that contributed to the protein–ligand interactions.

**Table 3:** Post-MD MM-GBSA binding free energy computation.

Complex	MM-GBSA Δ*G*_*bind*_ (kcal/mol)	Δ*G*_*bind*_ Coulomb (kcal/mol)	Δ*G*_*bind*_ Covalent (kcal/mol)	*ΔG*_*bind*_ Hbond (kcal/mol)	Δ*G*_*bind*_* Solv GB* (kcal/mol)	Δ*G*_*bind*_* Lipo* (kcal/mol)	Δ*G*_*bind*_* vdW* (kcal/mol)
PknG-**(**cyclo-(L-Pro-4-OH-L-Leu)**)**	− 21.1	− 7.6	0.9	− 0.6	8.2	− 5.9	− 16.16
PknG-co-crystallized (8ZC)	− 31.2	− 46.9	0.1	− 1.5	54.6	− 9.4	− 28.15
Pks13-vazabitide A	− 37.2	− 11.4	1.8	− 2.1	18.6	− 10.0	− 34.0
Pks13-co-crystallized (7IJ)	− 83.4	− 38.8	3.1	− 1.9	49.3	− 24.0	− 57.2

Δ*G*_*bind*_vdW, van der Waals contribution; Δ_*bind*_Covalent, covalent bonding contribution; Δ*G*_*bind*_Solv, polar contribution of solvation energy; Δ*G*_*bind*_Lipophilicity , lipophilicity energy contribution; Δ*G*_*bind*_Hbond, hydrogen bonding contribution; Δ*G*_*bind*_Coulomb, electrostatic interaction; Δ*G*_*bind*_, binding free energy.

### In-silico evaluation of the modified compound

A modified chemical compound was identified with the following smiles notation: c1cc(Cl)c(Cl)cc1C[C@@H](N(C(=O)[C@@H]2[NH3 +])C(=O)O[C@H](CC3)CCC23)CC(=O)NCc4c[nH +]cn4CCOCC. This modified compound showed an improved profile compared to vazabitide A. For instance, the percentage of human oral absorption increased to 71.28, the docking score decreased to − 13.2 kcal/mol, and the ΔG_*bind*_ was − 81.5 kcal/mol, which is significantly lower than vazabitide A (Table 4). All other parameters were within the acceptable range.

**Table 4:** Virtual screening of the modified compound against *M. tuberculosis* Pks13.

Complex	mol MW (170–725)	Dipole (1.0–12.5)	SASA	*QplogS (− 6.5 to 0.5)	PSA (7.0–200.0)	Volume	% Human oral absorption	XP GScore (kcal/mol)	MM-GBSA Δ*G*_*bind*_ kcal/mol
7VJT									
300405311 + 41735949 + 44455695	580.51	1.72	847.90	− 4.52	126.21	1658.13	71.28	− 13.2	− 81.5

**Figure 12:**
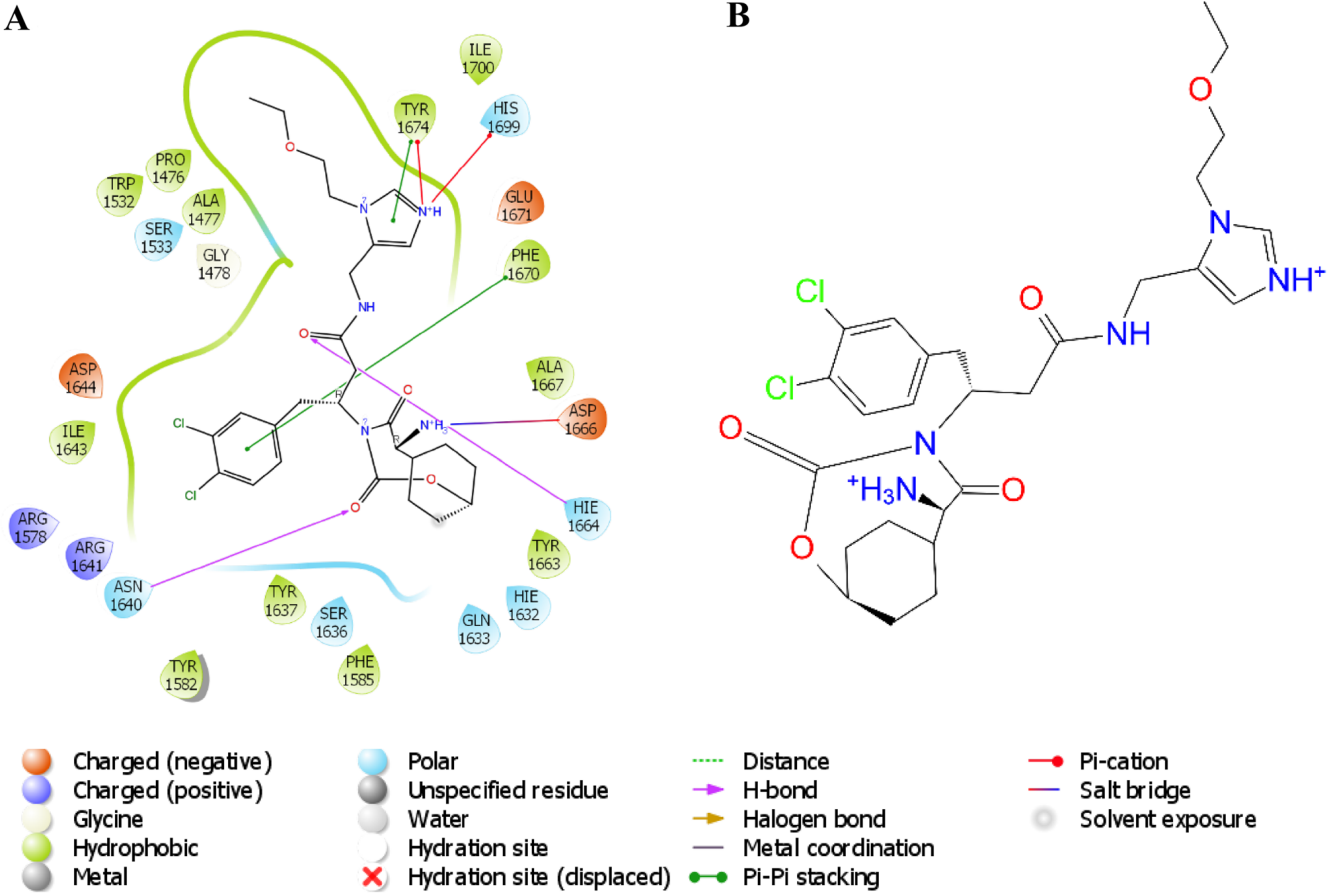
A concise overview of the interaction of the modified compound with Pks13 in two dimensions. (**A**) Superposition of the interaction of the modified compound with the binding pocket of Pks13. (**B**) Modified compound.

The modified compound's position and chemical properties enable several interactions that help stabilize the Pks13-ligand complex, as illustrated in Fig. 12B. The compound interacts with proximal amino acid residues of Pks13 in several ways, including hydrogen bonding with Asn1640 and He1644, hydrophobic interaction with Ala1477, Pro1476, Tyr1663, Ala1667, Phe1670, Tyr1674, Ile1700, Trp1532, Ile1643, Tyr1582, and Phe1585, polar interactions with Ser1533, His1699, Asn1640, Ser1636, Gln1633, He1632, and Hie1664, positive charges with Arg1641 and Arg1578, negative charges with Arg1641 and Arg1578, pi-cation interaction with Tyr1674 and His1699, pi-pi stacking with Phe1670 and Tyr1674, and salt bridge with Asp1666, as shown in Fig. 12A.

**Figure 13:**
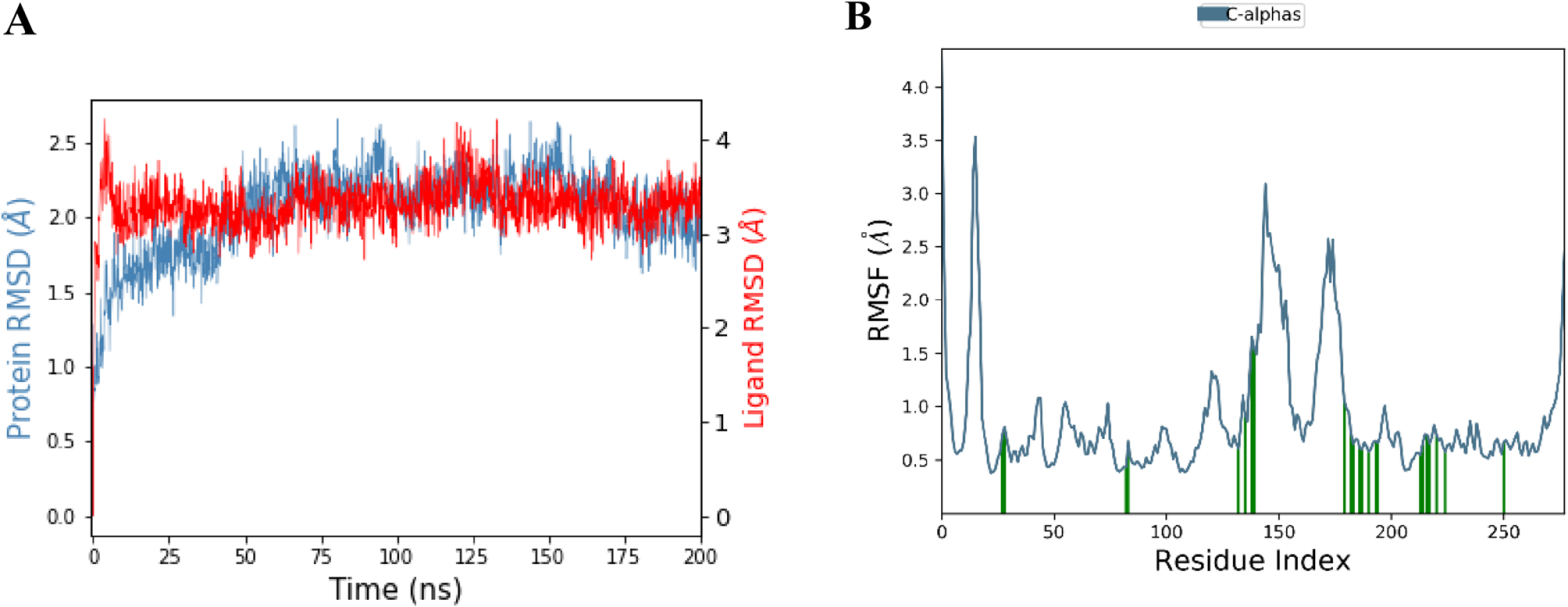
(**A**) RMSD of Pks13 Cα-atoms and the modified compound over a 200 ns simulation. (**B**) RMSF per residue of Pks13 in complex with the modified compound.

During a 200 ns molecular dynamics (MD) simulation, the Root Mean Square Deviation (RMSD) of Pks13 Cα-atoms was calculated. The results of the simulation showed that initially, the protein Cα-atoms gradually increased to approximately 2.1 Å within the first 50 ns. The Cα-atoms then stabilized at approximately 2.1 Å up to 150 ns, as shown in Fig. 13. Subsequently, the RMSD started to gradually decrease to approximately 1.8 Å up to 200 ns, indicating that the protein was stable throughout the simulation. Conversely, the ligand RMSD fluctuations were slightly above 3 Å during the MD simulations, indicating that the ligand was mobile within the protein's active site. The RMSF of Pks13 Cα-atoms showed that the residues did not fluctuate a lot except for the residues (10–20 and 170–180), which were constantly moving.

**Figure 14:**
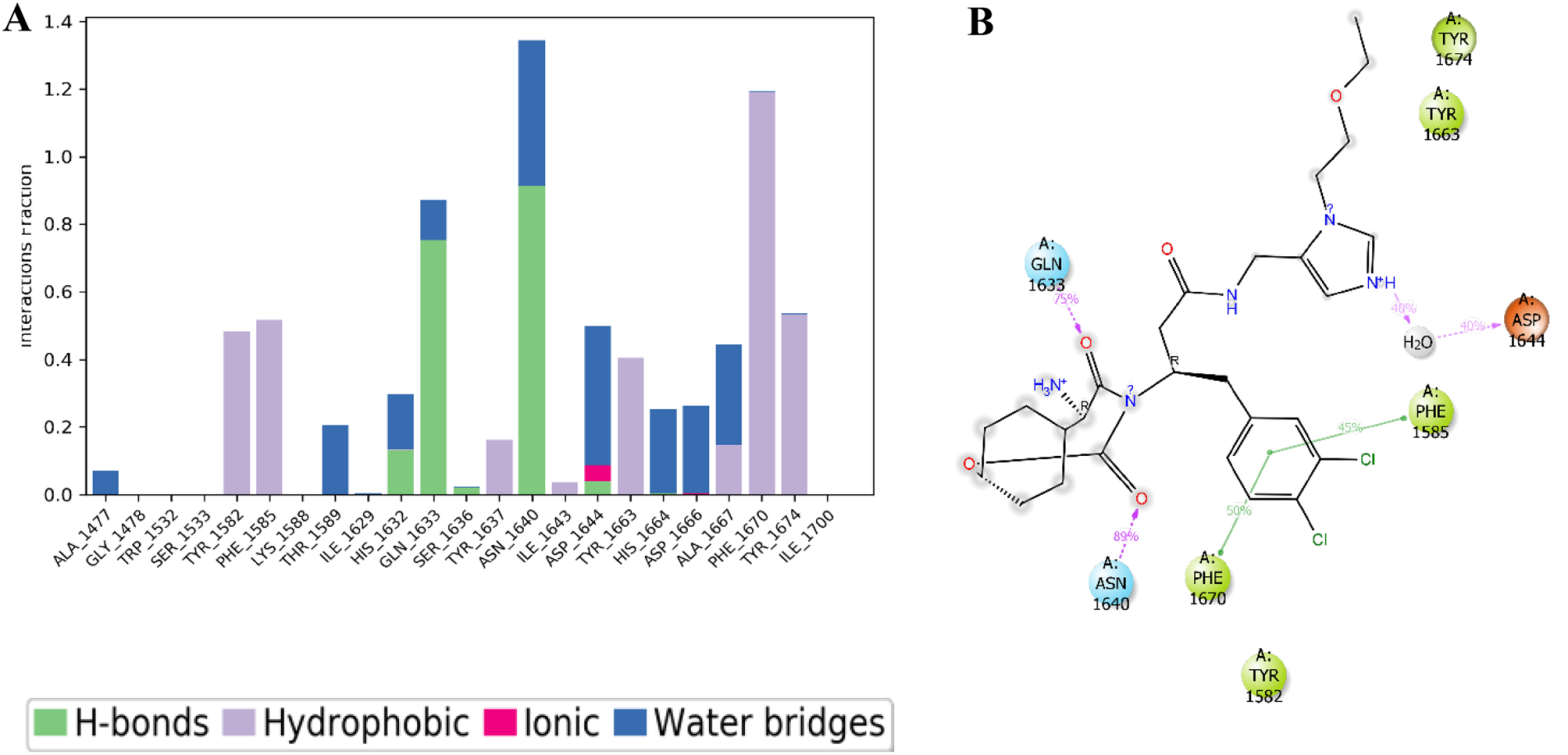
Interaction Fraction summary of Pks13-modified compound contacts. This graph is normalised by the total simulation time. (**A**) Histogram of the interaction fraction of the modified compound with Pks13. (**B**) Interactions that occurred for more than 30% of the 200 ns MD simulation.

During the 200 ns molecular dynamics simulation, it was observed that the carbonyl groups located at positions 4 and 9 of the modified compound had persistent hydrogen bonding interactions with polar amino acids Asn1640 and Gln1633, respectively, for a significant portion of the simulation time, as depicted in Fig. 14B. Additionally, the nitrogen atom located at position 32 formed water bridges with Asp1644 for 40% of the simulation time. The halide substituted benzene ring, which are at positions 31 and 28, contributed to hydrophobic interactions with nearby amino acid residues, namely Phe1670, Phe1585, and Tyr1582, throughout the simulation period. Notably, the substituted benzene ring also engaged in pi-pi stacking interactions with Phe1670 for 50% and Phe1585 for 45% of the simulation duration, highlighting the aromatic nature of the interaction. Overall, it can be concluded that hydrogen bonds, hydrophobic interactions, ionic interactions, and water bridges played a crucial role in influencing the stability of the protein–ligand complex (Fig. 14A).

The modified compound exhibited a stronger binding affinity to Pks13 as indicated by the MM-GBSA Δ*G*_*bind*_ of − 85.8 kcal/mol. The affinity is higher than co-crystallized (7IJ) and vazabitide A as shown in Tables 3 and 5. The Δ*G*_*bind*_ Coulomb and Δ*G*_*bind*_* vdW* significantly contributed to the strong bind properties observed. The modified compound also exhibited an Δ*G*_*bind*_*Lipophilicity*, which was more than that of the co-crystallized (7IJ) and vazabitide A.

**Table 5:** Post-MD MM-GBSA binding free energy computation.

Complex	MM-GBSA Δ*G*_*bind*_ (kcal/mol)	Δ*G*_*bind*_ Coulomb (kcal/mol)	Δ*G*_*bind*_ Covalent (kcal/mol)	$\Delta$*G*_*bind*_ Hbond (kcal/mol)	$\Delta$ *G*_*bind*_* Solv GB* (kcal/mol)	Δ*G*_*bind*_* Lipo* (kcal/mol)	Δ*G*_*bind*_* vdW* (kcal/mol)
Pks13-modified compound	− 85.8	− 69.0	0.4	− 1.1	64.8	− 22.5	− 54.5

Δ*G*_*bind*_vdW, van der Waals contribution; Δ_*bind*_Covalent, covalent bonding contribution; Δ*G*_*bind*_Solv, polar contribution of solvation energy; Δ*G*_*bind*_Lipophilicity, lipophilicity energy contribution; Δ*G*_*bind*_Hbond, hydrogen bonding contribution; Δ*G*_*bind*_Coulomb, electrostatic interaction; Δ*G*_*bind*_, binding free energy.

## Discussion

Gold mine tailings are composed of various fractions of mineral species, including nonessential elements such as Pb, As, as well as elements like Fe, Mg, Co, Mn, Cr, K, and Cn^45^. Research has shown that the acidic pH in gold tailings molecularly modifies the bioavailability and solubility of the heavy metals, creating an extreme environmental niche that selectively modulates microbial proliferation^46–48^. Therefore, the indigenous bacteria to the tailings must possess the molecular machinery that enables them to intimately associate with heavy metal transformation and tolerate the oxidative stress caused by heavy metals^49^. Various reports have shown the intimate interaction of *Bacillus *sp., *Acidithiobacillus *sp., *Arthrobacter *sp., *Pseudomonas *sp., *Microbacterium *sp., and Sp*hingomonas *sp with heavy metals^46,50,51^. Similarly, the bacteria identified in this study are actinobacteria, which are commonly found in different soil environments^52,53^. *Bacillus *sp was the most abundant culturable bacteria identified in South African gold mine tailings.

Earlier reports have shown that certain types of bacteria, such as *Bacillus *sp., *Micrococcus *sp., and *Pseudomonas *sp. can survive under abiotic stress^50,54–56^. The bacterial strains identified in this study are known to exhibit remarkable competence in alleviating heavy metal stress and thriving in low-pH environments. The molecular mechanisms underlying their unique metal acquisition and resistance determinants are well-characterized^49^. One such mechanism involves the extracellular secretion of surfactins/siderophores by *Bacillus *sp. These small molecules facilitate the removal of heavy metals from the surrounding environment, increase the bioavailability of water-insoluble nutrients, as a means of communication between bacterial cells and as an antimicrobial agent^57^. This is in accordance with the results from this report that demonstrate the capability of *Bacillus *sp*,* isolated from the heavy metal-rich environment, to produce multiple surfactin isomers. These isomers may potentially enhance the solubilization of heavy metals (Table S1).

A previous study illustrated that bacterial species that are closely related and possess higher homology in their biosynthetic gene cluster tend to have a lower chance of inhibiting each other^58^. Conversely, distant species in the same genus will likely suppress each other more fiercely^58^. The results of this study revealed that the crude extracts derived from *B. licheniformis*, *S. mycarofaciens*, and *B. subtilis* showed potent antimicrobial efficacy against *M. smegmatis* mc^2^155 and *M. aurum* A+, with *B. paralicheniformis* exhibiting activity only against *M. smegmatis* mc^2^155. Notably, only *B. subtilis* and *B. licheniformis* demonstrated efficacy against *M. tuberculosis* H37Rv. *Bacillus subtilis* and *Bacillus licheniformis* are well known to produce a lethal broad-spectrum antimicrobial arsenal that effectively inhibits both Gram-positive and Gram-negative bacteria by downregulating peptidoglycan synthesis^59^. Our results are supported by reports demonstrating that *B. licheniformis* CG1 produced bioactive metabolites that inhibited the growth of *M. smegmatis*^60,61^*.*

The current study yielded promising results, indicating that crude extracts obtained from *B. subtilis* and *B. licheniformis* exhibit antimycobacterial activity. To further investigate the finding, metabolite profiling was performed with the objective of tentatively identifying compounds responsible for activity (Fig. 2; Table S1). Microorganisms existing in extremophilic niches are known to have a higher probability of producing multifarious novel bioactive chemical classes^37^. Moreover, the study has also revealed various pathways associated with bioactivity through pathway enrichment analysis, such as secondary metabolites biosynthesis, as elaborated in Fig. 3 and Table S1. The identified pathways play an essential role in producing various bioactive metabolites. These pathways provide valuable insights into the metabolic capabilities of bacteria found in gold mine tailings. The knowledge from this investigation may aid in the hunt for novel antimycobacterial agents by examining biosynthetic functional gene clusters of the extremophilic bacteria. The results of this study corroborate earlier research that revealed that bacteria from extreme environments hold the potential to produce unique metabolites, as demonstrated by the discovery of several uncharacterised compounds, as shown in Fig. 2).

Earlier studies have shown a significant correlation between the Gram-positive *Bacillus* genus and the production of a diverse range of secondary metabolites. These include nonribosomal polyketides, nonribosomal lipopeptides, ribosomally synthesized and post-translationally modified peptides, and peptide-polyketide hybrid compounds^58,62^. The results of this study are consistent with previous reports indicating that *B. subtilis* and *B. licheniformis* produce cyclic lipopeptides and cyclic dipeptides, such as cyclo(proline-leucine), isomers of surfactin, and cyclo(L-Leu-L-4-Hyp), iturin derivatives^61^. Surfactins, cyclo(proline-leucine), and cyclo(L-Leu-L-4-Hyp) produced by various *Bacillus* species have been shown to possess significant permeabilization of microbial cell membranes and antimicrobial activity against both Gram-positive and Gram-negative bacteria^61^. While there is limited research specifically on the activity of the isomers of surfactin, such as surfactin C, surfactin A, and surfactin D, against *M. tuberculosis* H37Rv, some studies have investigated the activity of crude extracts containing surfactin against *Mycobacteri*a species^61^. The present study supports the previously reported findings that multiple isomers were tentatively identified in a crude extract of *Bacillus* sp. Additionally, the crude extract from *Bacillus* sp. exhibited potent activity against *M. tuberculosis* H37Rv. The study indicates that the strong inhibition observed in the crude extracts of *B. subtilis* and *B. licheniformis* may be attributed to an increased pore formation of the *Mycobacterium* cell membrane caused by different surfactin isoforms and other cyclic lipopeptides present in the crude extracts. The potent anti-mycobacterial efficacy against *M. smegmatis* mc^2^155, *M. aurum* A+, and *M. tuberculosis* H37Rv demonstrated by MICs from *B. subtilis* and *B. licheniformis* may have resulted from the synergistic effect of cyclic lipopeptides and cyclic dipeptides (Table S1).

Interaction mapping from molecular docking and MD simulations is crucial in revealing the significance of ligand binding towards the stability of a protein–ligand complex and the inhibition of a protein target. The HOMO–LUMO results in the study indicate that the compounds returned from the virtual screening are soft and highly reactive, as observed by the low docking scores. This study investigated the contribution of non-covalent interactions to the stability of PknG-cyclo-(L-Pro-4-OH-L-Leu) and Pks13-vazabitide A complexes during 200 ns MD simulations. The results revealed that several types of non-covalent interactions, including hydrogen bonding, water bridges, ionic interactions, and hydrophobic interactions, played a critical role in restricting major conformational changes of the protein–ligand complexes. According to the HOMO–LUMO results, the interaction with the nitrogen rings was as expected. Specifically, key residues, including GLU167, ASN1640, and ASP1644, formed hydrogen bonds with vazabitide A, which occurred for more than 30% of the 200 ns simulation time and were crucial in stabilizing the Pks13-vazabitide A complex.

The analysis conducted after the molecular dynamics simulation involved calculating the protein–ligand binding free energies based on MD simulation trajectories. The comparison of the MM/GBSA binding free energy of vazabitide A and cyclo-(L-Pro-4-OH-L-Leu) supports the RMSD profiles from the two Pks13 complexes, which showed excellent stability to the co-crystallized ligand (7IJ). Vazabitide A also interacted with the SER1533 of Pks13 thioesterase domain (TE) through water bridges. The TE domain is an acyltransferase that is responsible for cleaving the thioester bond and forming an ester bond between the mycolic β-ketoester and the hydroxyl group of Ser1533 of the TE domain to form a trehalose monomycolate ketone. Studies have revealed that blocking the TE domain of Pks13 abolished the biogenesis of mycolic acids and consequently inhibited the growth of *M. tuberculosis*^26^*.* Noteworthy, vazabitide A is a natural compound that can be further modified to enhance its important properties, such as affinity, toxicity, and activity. In this study, the RMSD generated from the trajectory of PknG Cα-atoms complexed with cyclo-(L-Pro-4-OH-L-Leu) in a 200 ns simulation revealed that cyclo-(L-Pro-4-OH-L-Leu) demonstrated a relatively better binding dynamics than the co-crystallized ligand during the first 100 ns. These results provide insight into the relatively favourable binding mode of cyclo-(L-Pro-4-OH-L-Leu) to PknG during the initial 100 ns. The interaction analysis suggests that some binding characteristics may be conserved while others are increased during scaffold modification against Pks13.

During the simulation, it was observed that the modified compound's carbonyl groups at positions 4 and 9 formed persistent hydrogen bonding interactions with polar amino acids ASN1640 and GLN1633, respectively (Fig. 14). The protein–ligand interaction indicated that the electrostatic and polar interactions between the carbonyl groups and amino acids were strong enough to contribute to the stability of the Pks13-modified compound-complex. Moreover, the substituted benzene ring with two chlorine atoms contributed to the compound's lipophilicity and binding affinity in the hydrophobic active site of Pks13 (Fig. 14). The nitrogen on the imidazole ring at position 32 of the modified compound formed water bridges with ASP1644, indicating polar interactions that were crucial in stabilizing the Pks13-modified compound-complex. Previous literature has reported various microbial activities of structural motifs with an imidazole ring. These findings offer an alternative perspective for developing new antimycobacterial scaffolds with improved efficacy and selectivity^63–65^.

## Conclusion

In this study, the tailings of South African gold mines are a promising source of bacteria that produce secondary metabolites with antimycobacterial properties. Three bacterial strains (*B. licheniformis*, *S. mycarofaciens*, and *B. subtilis*) isolated from this metal-rich environment were found to produce broad-spectrum secondary metabolites that can inhibit *M. smegmatis* mc^2^155, *M. aurum* A+, and *M. tuberculosis* H37Rv. *Bacillus subtilis* and *B. licheniformis* produced broad-spectrum antimicrobial cyclic lipopeptides such as surfactin and iturin derivatives. These bioactive molecules may have contributed to potent inhibitory activity against *M. tuberculosis* H37Rv. In addition, the HPLC-qTOF detected unknown compounds from *B. subtilis* and *B. licheniformis*, suggesting the production of novel compounds. The findings provide valuable insights into the metabolic potential of this *B. subtilis* group of microorganisms found in gold mine tailings. Moreover, it highlights the potential targets for the development of antibiotics. This may prove crucial for future research and advancement in understanding the versatile nature of *B. subtilis* and *B. licheniformis* and their ability to produce antimicrobial agents. Vazabitide A interacted with Ser1533 of the TE domain of Pks13. Vazabitide A exhibited an MM-GBSA Δ*G*_*bind*_ − 37.2 kcal/mol, which was less than the control co-crystallized ligand, and this led to the modification of Vazabitide A to increase affinity towards Pks13. The modification increased the MM-GBSA Δ*G*_*bind*_ to − 85.8 kcal/mol. The findings shed light on the interaction mapping of protein–ligand complexes, which may have implications for developing novel therapeutic agents targeting PknG and Pks13. Such developments could help reduce expenses and save time spent on extraction, purification, and retesting processes.

### Supplementary Information

Supplementary Information 1.

Supplementary Information 2.

## Data Availability

The datasets generated during and/or analysed during the current study are available from the corresponding author upon reasonable request.
