# Supplementary material for: Molecular docking, molecular dynamics simulations and binding free energy studies of interactions between Mycobacterium tuberculosis Pks13, PknG and bioactive constituents of extremophilic bacteria
Source: Sci Rep. 2024 Mar 21;14:6794. doi: 10.1038/s41598-024-57124-9 (PMC10957976; doi:10.1038/s41598-024-57124-9)
Supplement: Supplementary file 1 — Supplementary Information 1. [file 41598_2024_57124_MOESM1_ESM.docx]

## **Supplementary File 1**

**Molecular docking, molecular dynamics simulations and binding free energy studies of interactions between *Mycobacterium tuberculosis* Pks13, PknG and bioactive constituents of extremophilic bacteria**

**Kudakwashe Nyambo^1^, Kudzanai Ian Tapfuma^1^, Francis Adu-Amankwaah^1^, Lucinda Baatjies^1^,** **Idah Sithole Niang^2^, Liezel Smith^1^, Krishna Kuben Kuben Govender^4,5^, Mkhuseli Ngxande^3^, Daniel J Watson^6^, Lubbe Wiesner^6^,** **and Vuyo Mavumengwana^1,^ ***

^1^*DSI-NRF Centre of Excellence for Biomedical Tuberculosis Research; South African Medical Research Council Centre for Tuberculosis Research; Division of Molecular Biology and Human Genetics, Faculty of Medicine and Health Sciences, Stellenbosch University, Cape Town; KN:* [*knyambo@sun.ac.za*](mailto:knyambo@sun.ac.za)*; K.I.T: kudzanait@sun.ac.za; LB: lbaatjies@sun.ac.za; LS: liezels@sun.ac.za; VM:* [*vuyom@sun.ac.za*](mailto:vuyom@sun.ac.za)

^2^*Department of Biotechnology and Biochemistry, University of Zimbabwe, B064, Mount Pleasant, Harare, Zimbabwe;* [*sitholeidah2015@gmail.com*](mailto:sitholeidah2015@gmail.com)

*^3^Computer Science Division, Department of Mathematical Sciences,* *Faculty of Science University of Stellenbosch, Matieland, South Africa;* [*ngxandem@sun.ac.za*](mailto:ngxandem@sun.ac.za)

*^4^Department of Chemical Sciences, University of Johannesburg, P. O. Box 17011, Doornfontein Campus, 2028, Johannesburg, South Africa;* [*krishnag@uj.ac.za*](mailto:krishnag@uj.ac.za)

*^5^National Institute for Theoretical and Computational Sciences (NITheCS), South Africa*

*^6^Division of Clinical Pharmacology, Department of Medicine, Faculty of Health Sciences, University of Cape Town; D.J.W:* [*daniel.watson@uct.ac.za*](mailto:daniel.watson@uct.ac.za)*; L.W: lubbe.wiesner@uct.ac.za*

***** Corresponding author.

*Email address*: [*vuyom@sun.ac.za*](mailto:vuyom@sun.ac.za)*;* Tel: +27 718502949

**Table S1.** Tentatively identified metabolites present in bacterial extracts.

| **Sample** | **Classification** | **Formula** | **Tentative Identification** | **Precursor m/z**  **[M+H]^+^** | **m/z Error**  **(ppm)** |
| --- | --- | --- | --- | --- | --- |
| ***S. mycarofaciens*** |  |  |  |  |  |
|  | Alpha amino acids and derivatives | C_7_H_10_N_2_O_2_ | cyclo-L-Prolylglycine | 155.0811 | 0.000404 |
|  | Alpha amino acids and derivatives | C_14_H_16_N_2_O_3_ | Maculosin | 261.1234 | 0.8334 |
|  | Aminocyclitol glycosides | C_19_H_37_N_5_O6 | Istamycin C1 | 432.2802 | 0.7802 |
|  | Alpha amino acids and derivatives | C_11_H_18_N_2_O_2_ | L,L-Cyclo(leucylprolyl) | 211.1437 | 0.8737 |
|  | Alpha amino acids and derivatives | C_14_H_16_N_2_O_2_ | cyclo-(L-Phe-L-Pro) | 245.1283 |  |
|  |  | C_9_H_11_NO_3_ | unknown | 182.0815 | -1.8241 |
|  | Alpha amino acids and derivatives | C_11_H_14_N_4_O_2_ | NPA006809 | 235.1191 | -0.6317 |
|  |  | C_11_H_16_N_2_O_2_ | Unknown | 209.1278 | 3.1434 |
|  | Organic acids and derivatives | C_12_H_21_N_3_O_4_ | Vazabitide A | 272.1614 | -3.3833 |
|  | Glycosides | C_12_H_13_N_5_O_3_ | 5’- deoxytoyocamycin | 276.1082 | 3.3286 |
|  | Organoheterocyclic | C_15_H_21_N_3_O_2_ | Physostigmine | 276.1714 | -2.7133 |
|  |  | C_14_H_25_NO_6_ | unknown | 304.1762 | -2.4277 |
|  | Organic acids and derivatives | C_10_H_18_N_2_O_3_ | Geralcin E | 215.1393 | -1.3127 |
|  | Lipids and lipid-like molecules | C_11_H_20_N_2_O_3_ | α-Methyldethiobiotin | 229.1558 | -4.9573 |
|  | Alpha amino acids and derivatives | C_10_H_16_N_2_O_2_ | cyclo(L-Pro-L-Val) | 197.1294 | -4.8225 |
|  | Alpha amino acids and derivatives | C_11_H_18_N_2_O_3_ | Cyclo(2-hydroxy-Pro-R-Leu) | 227.14 | -4.3386 |
|  | Alpha amino acids and derivatives | C_14_H_16_N_2_0_3_ | Cyclo(D-Pro-L-Tyr) | 261.1239 | -2.0422 |

**Table S1.** Tentatively identified metabolites present in bacterial extracts. Cont..

| **Sample** | **Classification** | **Formula** | **Tentative Identification** | **Precursor**  **m/z**  **[M+H]^+^** | **m/z Error (ppm)** |
| --- | --- | --- | --- | --- | --- |
| ***S. mycarofaciens*** |  |  |  |  |  |
|  | Alpha amino acids and derivatives | C_11_H_18_N_2_O_2_ | Cyclo(D)-Pro-(D)-Ile | 211.1447 | -2.8348 |
|  | Alpha amino acids and derivatives | C_14_H_16_N_2_O_2_ | Cyclo(D)-Pro-(D)-Phe | 245.1285 | -0.1876 |
|  | Alpha amino acids and derivatives | C_11_H_14_N_4_O_2_ | JBIR-75 | 235.1191 | 0.8691 |
|  | Alpha amino acids and derivatives | C_19_H_36_N_2_O_5_ | Lipoxamycin | 373.2704 | 0.7704 |
|  | Alpha amino acids and derivatives | C_10_H_16_N_2_O_2_ | 2-amino-N-(2’-(cyclohex-2’’-enyl)acetyl)acetimide | 197.1291 | 0.8791 |
|  |  |  |  |  |  |
| ***B. subtilis*** |  |  |  |  |  |
|  | Alpha amino acids and derivatives | [C_11_H_18_N_2_O_3_](https://pubchem.ncbi.nlm.nih.gov/#query=C11H18N2O3) | Cyclo-(L-Pro-4-OH-L-Leu) | 227.1387 | 1.4102 |
|  | Alpha amino acids and derivatives | C_11_H_18_N_2_O_2_ | Cyclo(proline-leucine) | 211.1436 | 0.0005043 |
|  | Alpha amino acids and derivatives | C_7_H_10_N_2_O_2_ | cyclo-L-Prolylglycine | 155.0812 | 0.000304 |
|  | Indoles | C_11_H_9_NO_2_ | Indole-3-acrylic acid | 188.0704 | 0.000205 |
|  | Alpha amino acids and derivatives | C_10_H_16_N_2_O_2_ | cyclo(L-Pro-L-Val) | 197.128 | 0.0004542 |
|  | Alpha amino acids and derivatives | C_14_H_16_N_2_O_2_ | cyclo-(L-Phe-L-Pro) | 245.128 | 1.8606 |
|  | Cyclic lipopeptide | [C_76_H_117_N_11_O_20_](https://pubchem.ncbi.nlm.nih.gov/#query=C72H110N12O20) | Plipastatin | 1505.94 | 4.5387 |
|  | Cyclic depsipeptides | C_51_H_89_N_7_O_13_ | Surfactin A | 1008.658 | 3.9654 |

**Table S1.** Tentatively identified metabolites present in bacterial extracts. cont..

| **Sample** | **Classification** | **Formula** | **Tentative Identification** | **Precursor m/z**  **[M+H]^+^** | **m/z Error (ppm)** |
| --- | --- | --- | --- | --- | --- |
| ***B. subtilis*** |  |  |  |  |  |
|  | Cyclic lipopeptide | [C_74_H_114_N_12_O_20_](https://pubchem.ncbi.nlm.nih.gov/#query=C72H110N12O20) | Plipastatin | 1492.4058 | 0.6058 |
|  | Cyclic depsipeptides | C_53_H_95_N_7_O_14_ | Gageostatin B | 1054.704 | -2.8688 |
|  | Cyclic depsipeptides | [C_52_H_91_N_7_O_13_](https://pubchem.ncbi.nlm.nih.gov/#query=C52H91N7O13) | Surfactin B | 1022.676 | 4.1201 |
|  | Cyclic depsipeptides | C_53_H_94_N_8_O_12_ | Surfactin C1 | 1036.69 | 0.3245 |
|  | Cyclic depsipeptides | C_53_H_89_N_13_O_8_ | Surfactin D | 1036.703 | -0.0161 |
| **(*B. licheniformis*)** |  |  |  |  |  |
|  | Valine and derivatives | C_5_H_11_NO_2_ | L-Valine | 118.0858 | 3.8863 |
|  |  | C_11_H_19_N_3_O_2_ | Unknown | 226.1555 | -2.2061 |
|  | Alpha amino acids and derivatives | C_12_H_18_N_4_O_2_ | Cis-cyclo-(His,Leu) | 251.1511 | -3.3889 |
|  |  | C_12_H_23_N_3_O_2_ | Unknown | 242.1862 | 3.0860 |
|  |  | C_32_H_43_N_5_O_8_ | Unknown | 626.3157 | 4.3815 |
|  | Alpha amino acids and derivatives | C_10_H_16_N_2_O_2_ | Cyclo(L-Pro-L-Val) | 197.1286 | -0.7434 |
|  |  | C_20_H_33_N_5_O_7_ | unknown | 456.2437 | 3.4593 |
|  | Alpha amino acids and derivatives | C_11_H_18_N_2_O_3_ | Cyclo(4-hydroxy-R-Pro-S-Leu) | 227.1396 | -2.5697 |
|  | Alpha amino acids and derivatives | C_14_H_16_N_2_O_3_ | Cyclo(D-6-Hyp-L-Phe) | 261.1232 | 0.6489 |

**Table S1**. Tentatively identified metabolites present in bacterial extracts. Cont.

| **Sample** | **Formulae** | **Classification** | **Tentative Identity** | **Precursor m/z**  **[M+H]^+^** | **m/z Error**  **(ppm)** |
| --- | --- | --- | --- | --- | --- |
| **(*B. licheniformis*)** |  |  |  |  |  |
|  | C_11_H_18_N_2_O_2_ | Alpha amino acids and derivatives | Cyclo(Pro-Leu) | 211.1446 | -2.3589 |
|  | C_12_H_14_N_2_O |  | Unknown | 203.1175 | 1.9272 |
|  | C_10_H_16_N_2_O_4_ |  | Unknown | 229.1181 | 0.8040 |
|  | C_16_H_29_N_3_O_6_ |  | Unknown | 360.2137 | -2.1935 |
|  | C_29_H_82_N_34_O_8_ |  | Unknown | 1035.713 | -0.2300 |
|  | C_11_H_19_N_5_O_2_ | Alpha amino acids and derivatives | Cyclo(D-Arg-L-Pro) | 254.1624 | -4.9326 |
|  | C_11_H_19_N_3_O_2_ |  | Unknown | 226.1555 | -2.2061 |
|  | C_12_H_18_N_4_O_2_ | Alpha amino acids and derivatives | Cis-cyclo-(His,Leu) | 251.1511 | -3.3889 |
|  | C_53_H_89_N_13_O_8_ | Cyclic depsipeptides | Surfactin D | 1036.703 | -0.0161 |
|  | C_53_H_94_N_8_O_12_ | Cyclic depsipeptides | Surfactin_C1 | 1036.69 | 0.3245 |
|  | [C_52_H_91_N_7_O_13_](https://pubchem.ncbi.nlm.nih.gov/#query=C52H91N7O13) | Cyclic depsipeptides | Surfactin B | 1022.676 | 4.1201 |
|  | C_8_H_16_N_4_O_3_ | N-acyl-L-alpha-amino acids | (2S)-2-acetamido-5-guanidino-valeric acid | 217.129 | 2.3912 |
|  | C_14_H_16_N_2_O_2_ | Dipeptides | (3s,8ar)-3-benzyl-1-hydroxy-3h,6h,7h,8h,8ah-pyrrolo[1,2-a]pyrazin-4-one | 245.1282 | 1.0413 |
|  | C_16_H_22_O_4_ | Sesquiterpenoids | mochiquinone | 279.1594 | -1.1303 |

| 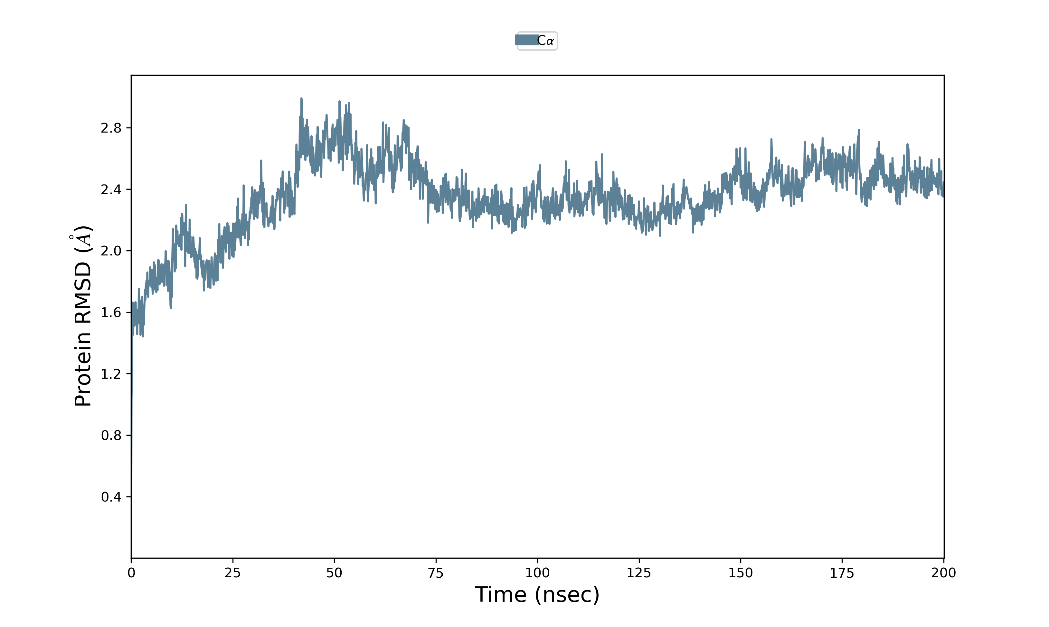  **A.** | **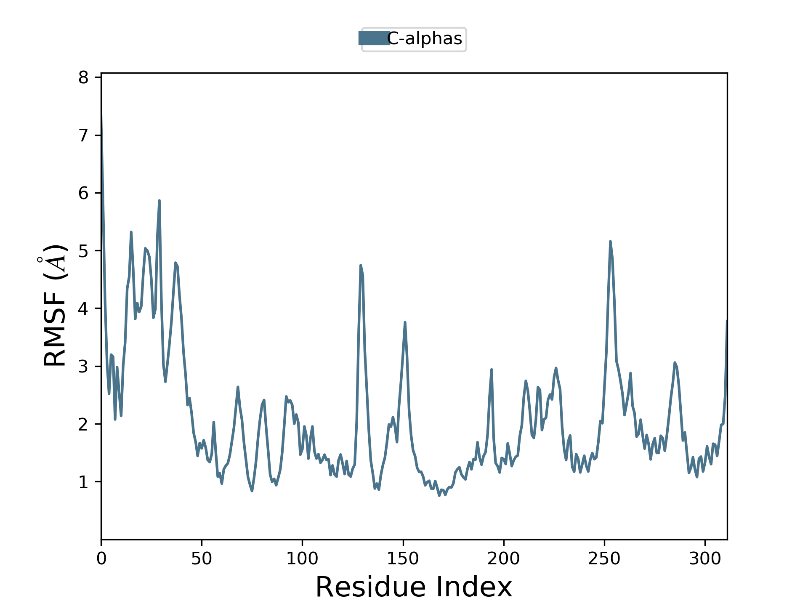**  **B.** |
| --- | --- |

**Figure S1. (A)** RMSD PknG Cα-atoms over a period of 200 ns MD simulation and **(B)** RMSD for PknG Cα-atoms


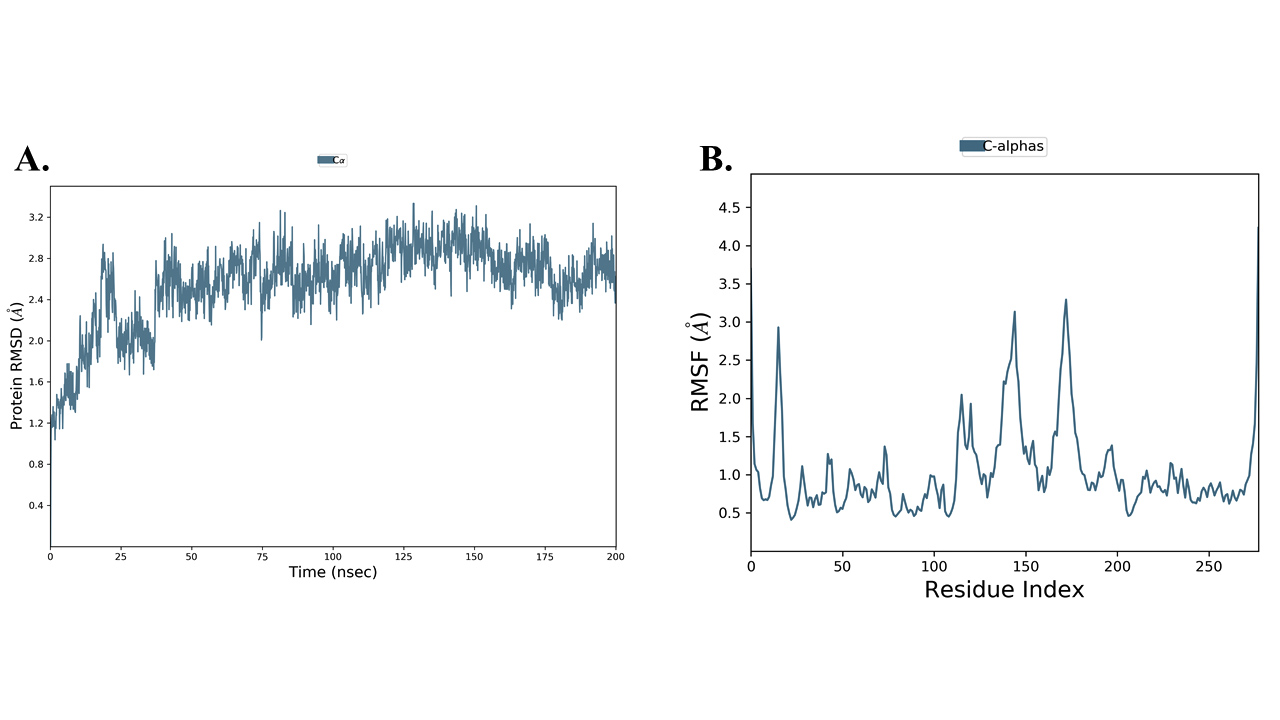


**Figure S2. (A)** RMSD Pks13 Cα-atoms over a period of 200 ns MD simulation and **(B)** RMSF of Pks13 Cα-atoms


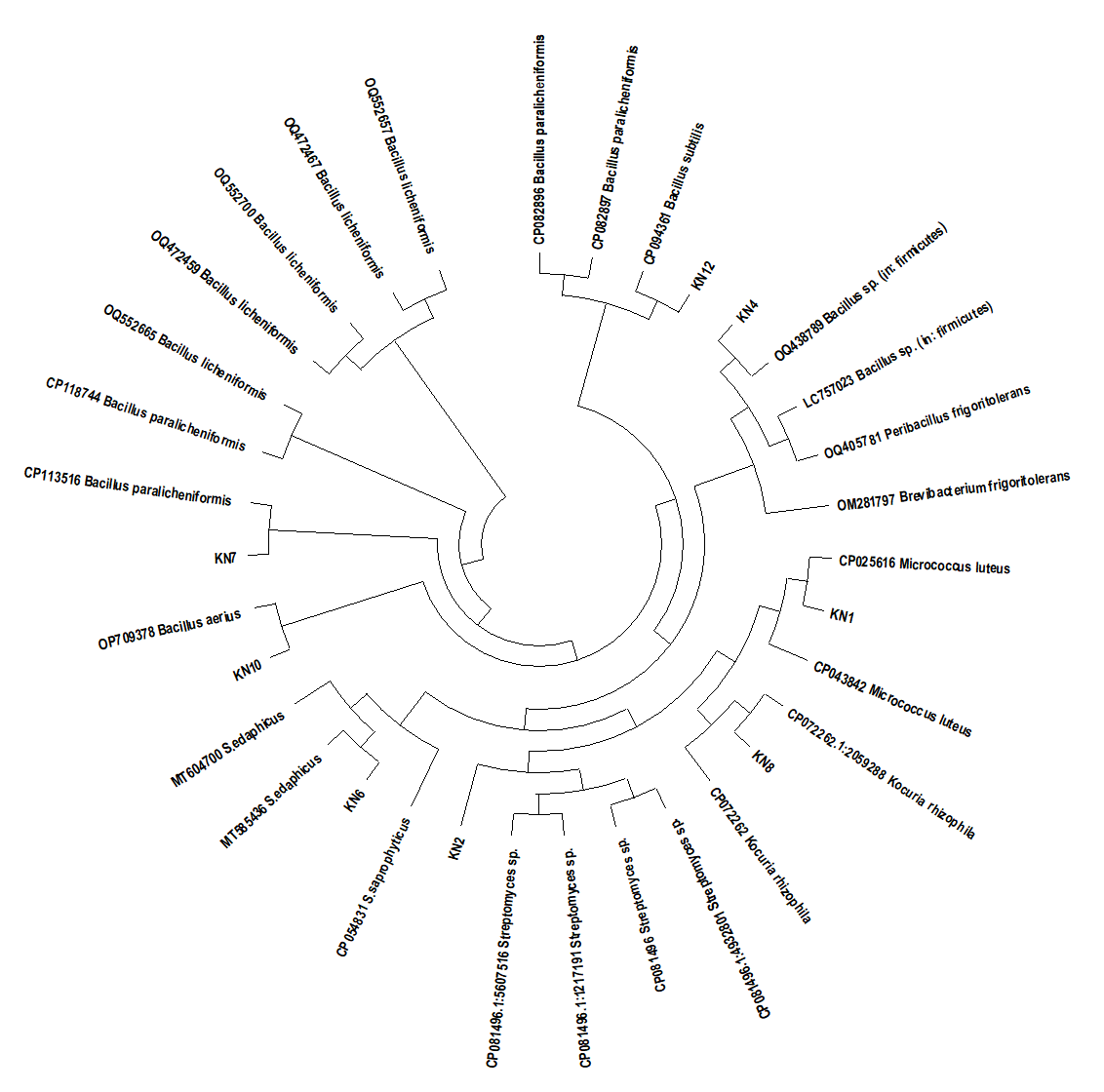


**Figure S3.** Phylogenetic position of 8 isolated bacterial strains. A maximum likelihood (ML) tree was constructed based on 16 S rRNA sequences of good quality.

| 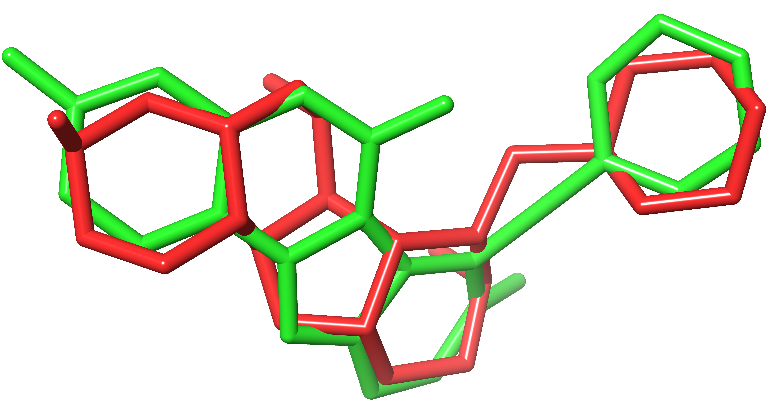  A | **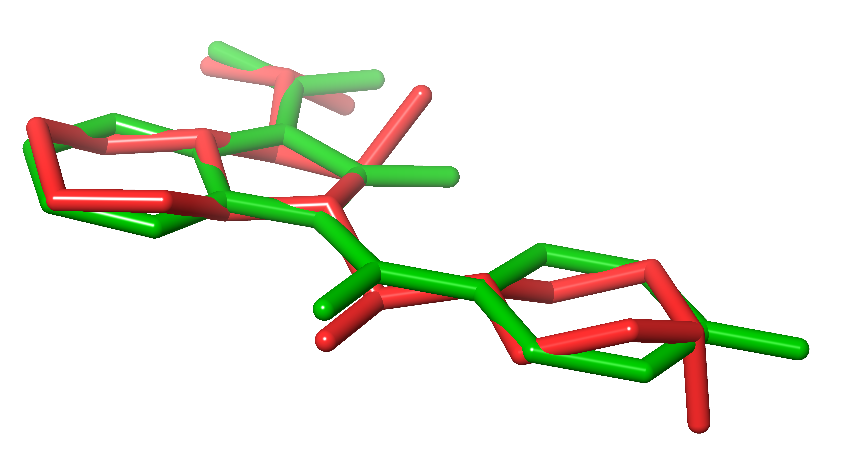**  B |
| --- | --- |

**Figure S4**. **(A)** The RMSD of the co-crystalized ligand (7IJ) vs the docked co-crystalized ligand. **(B)** The RMSD of the native co-crystalized ligand (8ZC) vs the docked co-crystalized ligand (8ZC). Green represents the native co-crystalized ligand while red represents docked co-crystalized ligand.

**Table S2**. Computed orbital energies of the tentatively identified bacterial compounds.

| **Compounds** | $\boldsymbol{E}$**_HOMO_** | $\boldsymbol{E}$**_LUMO_** | **Energy gap** $\boldsymbol{\Delta}\boldsymbol{E}$ | **Ionisation energy^a^** | **Electron Affinity^b^** | **µ** | **ɲ** | **s** | **ꭕ** | **Ꞷ** |
| --- | --- | --- | --- | --- | --- | --- | --- | --- | --- | --- |
| Vazabitide A | $-$0.31 | $-$0.02 | 0.29 | 0.31 | 0.02 | $-$0.16 | 0.15 | 6.83 | 0.16 | 0.09 |
| Cyclo(Pro-Leu) | $-$0.31 | $-$0.01 | 0.30 | 0.31 | 0.01 | $-$0.16 | 0.15 | 6.72 | 0.16 | 0.09 |
| Maculosin | $-$0.28 | $-$0.01 | 0.27 | 0.28 | 0.01 | $-$0.15 | 0.13 | 7.51 | 0.15 | 0.08 |
| 5'-Deoxytoyocamycin | $-$0.29 | $-$0.03 | 0.26 | 0.29 | 0.03 | $-$0.15 | 0.13 | 7.65 | 0.16 | 0.10 |
| (3R,8aR)-3-benzyl-2,3,6,7,8,8a-hexahydropyrrolo[1,2-a]pyrazine-1,4-dione | $-$0.30 | $-$0.01 | 0.29 | 0.30 | 0.01 | $-$0.16 | 0.14 | 6.94 | 0.16 | 0.08 |
| NPA006809 | $-$0.27 | $-$0.04 | 0.23 | 0.27 | 0.04 | $-$0.16 | 0.12 | 8.61 | 0.16 | 0.10 |
| Cyclo(2-hydroxy-Pro-R-Leu) | $-$0.32 | $-$0.01 | 0.30 | 0.32 | 0.01 | $-$0.17 | 0.15 | 6.59 | 0.17 | 0.09 |
| cyclo-(L-Pro-4-OH-L-Leu) | $-$0.31 | $-$0.01 | 0.29 | 0.31 | 0.01 | $-$0.16 | 0.15 | 6.79 | 0.1 | 0.09 |

chemical potential (µ), chemical hardness (ɲ), chemical softness (s), electronegativity (ꭕ) and electrophilic index (Ꞷ).

$ꭕ= -\mu$ (S1)

$Ꞷ= \frac{\mu^{2}}{2ɲ}$ (S2)

| 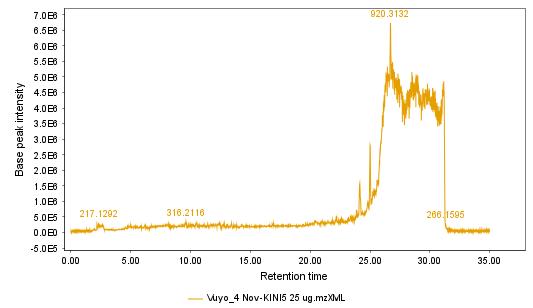  **A.** | 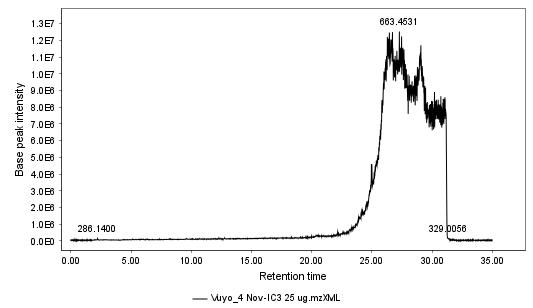  **B.** |
| --- | --- |
| 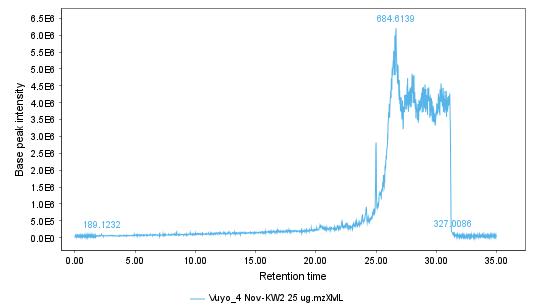  **C.** |  |

**Figure S5.** LC-MS-QTOF detection of natural products. **(A)** Base peak intensity corresponding to *B. subtilis.* **(B)** Base peak intensity corresponding to *S. mycarofaciens*. **(C)** Base peak intensity corresponding *B. licheniformis.*

| 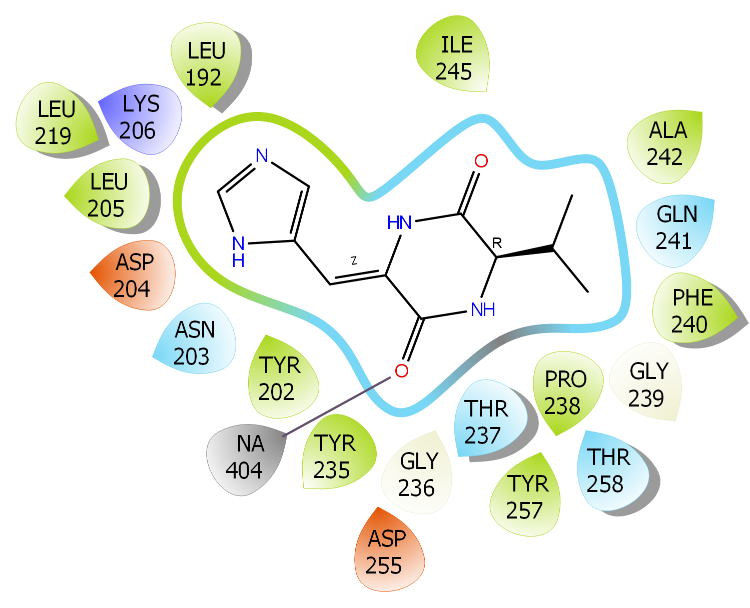  **A.** | 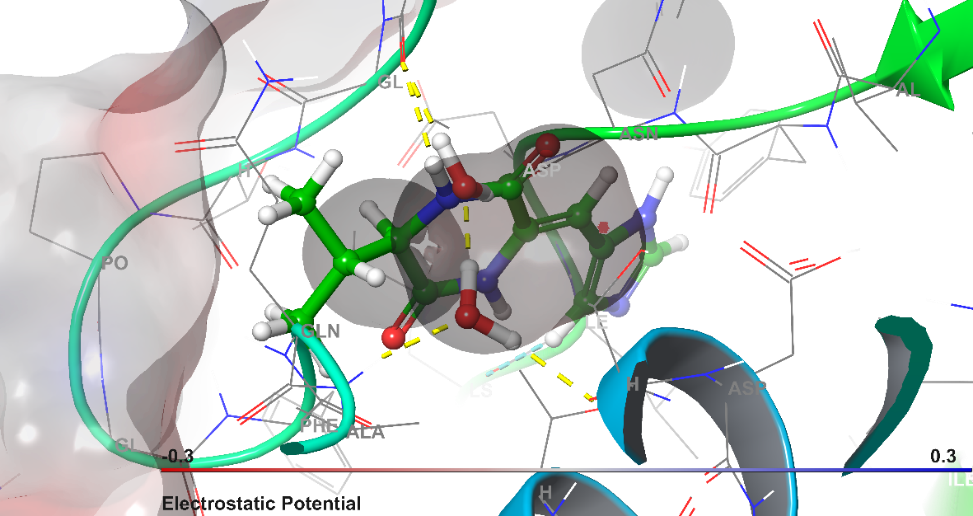  **B.** |
| --- | --- |
| 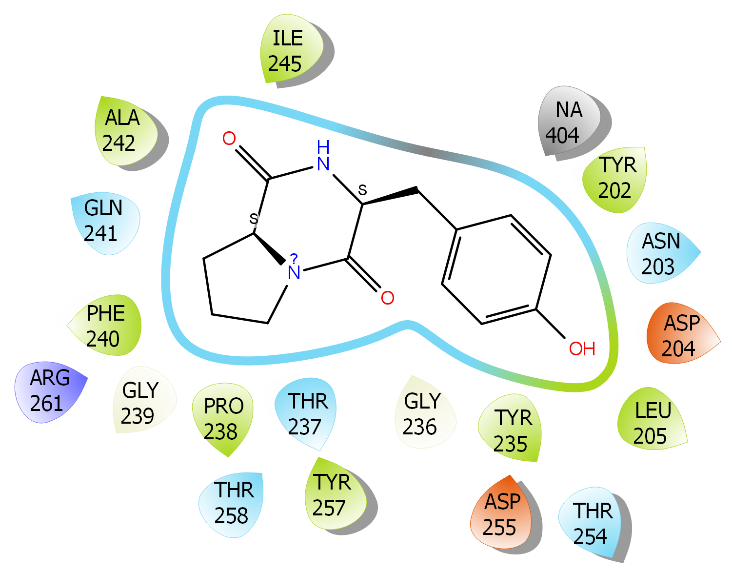  **C.** | 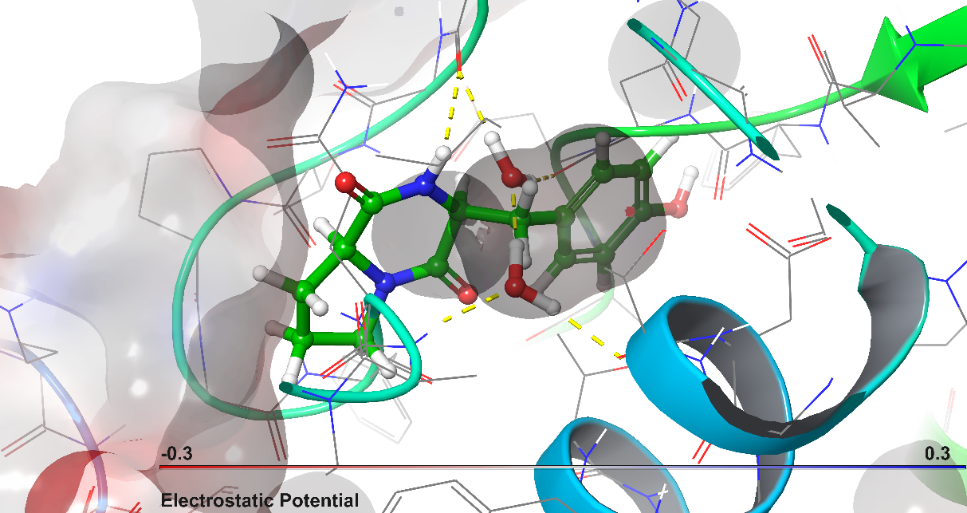  **D.** |

**Figure S6.** A concise superposition of the interaction of protein and ligand (A) 2D representation of NPA006809 in the binding pocket of PknG (B) 3D representation of NPA006809 in the binding pocket of PknG (C) 2D representation of maculosin in the binding pocket of PknG (D) 3D representation of maculosin in the binding pocket of PknG

| **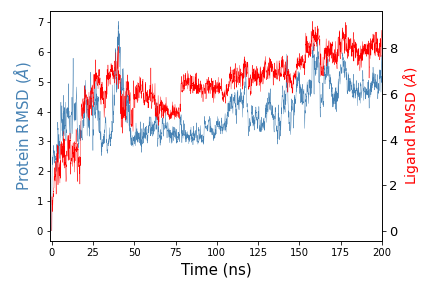**  **A.** | **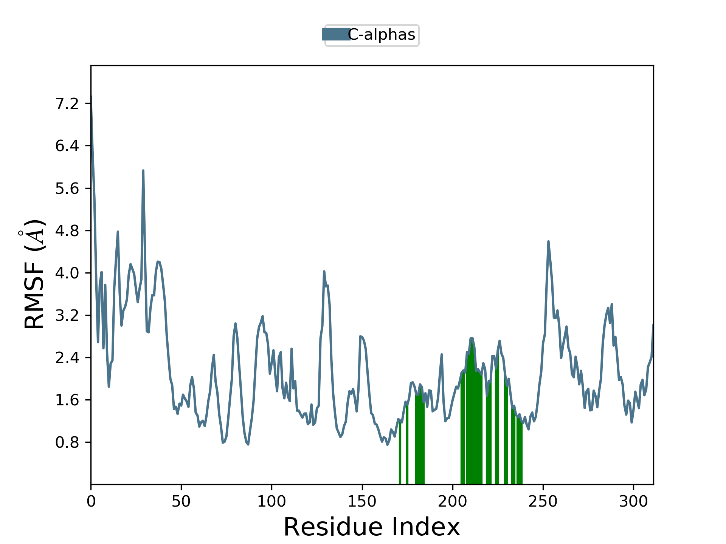**  **B.** |
| --- | --- |
| 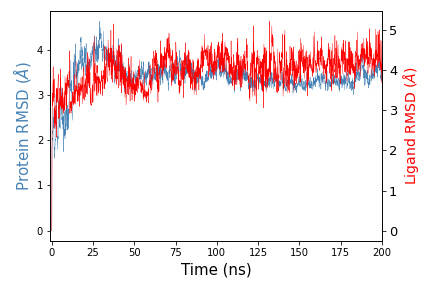  **C.** | 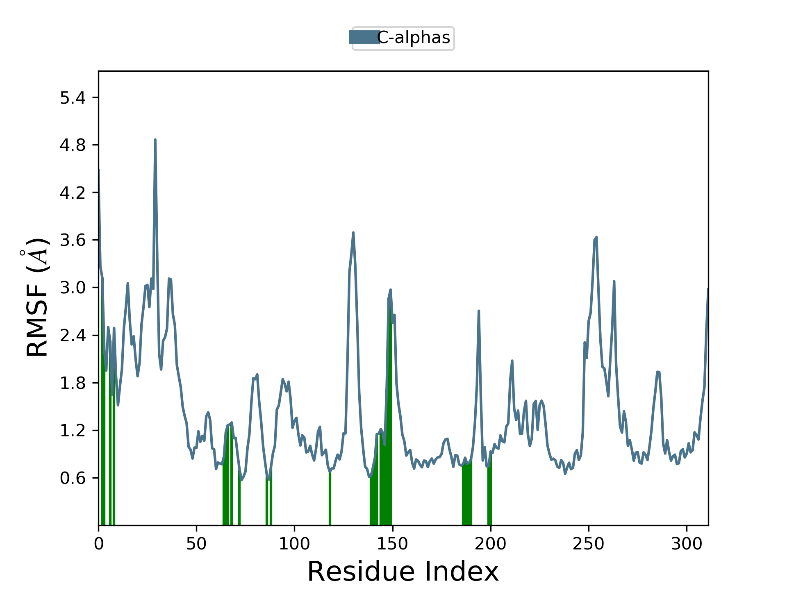  **D.** |

**Figure S7. (A)** RMSD of PknG Cα-atoms and the NPA006809 over a 200 ns simulation. **(B)** RMSF per residue of PknG in complex with NPA006809. **(C)** RMSD of PknG Cα-atoms and the maculosin over a 200 ns simulation. **(D)** RMSF per residue of PknG in complex with maculosin.
